# Maximum size and magnitude of injection-induced slow slip events

**DOI:** 10.1126/sciadv.adq0662

**Published:** 2025-05-07

**Authors:** Alexis Sáez, François Passelègue, Brice Lecampion

**Affiliations:** ^1^ Geo-Energy Lab - Gaznat Chair, École Polytechnique Fédérale de Lausanne (EPFL), Lausanne, Switzerland.; ^2^ Géoazur, Université Côte d’Azur, Nice, France.

## Abstract

Fluid injections can induce aseismic slip, resulting in stress changes that may propagate faster than pore pressure diffusion, potentially triggering seismicity at substantial distances from injection wells. Constraining the maximum extent of these aseismic ruptures is, thus, important for better delineating the influence zone of injections concerning their seismic hazard. Here, we derive a scaling relation based on rupture physics for the maximum size of aseismic ruptures, accounting for fluid injections with arbitrary flow rate histories. Moreover, on the basis of mounting evidence that the moment release during these operations is often predominantly aseismic, we derive a scaling relation for the maximum magnitude of aseismic slip events. Our theoretical predictions are consistent with observations over a broad spectrum of event sizes, from laboratory to real-world cases, indicating that fault zone storativity, background stress change, and injected fluid volume are key determinants of the maximum size and magnitude of injection-induced slow slip events.

## Teaser

Scaling laws reveal how subsurface fluid injections control the maximum size and magnitude of induced aseismic slip events.

## INTRODUCTION

A growing body of observations suggests that a substantial part of the deformation induced by subsurface fluid injections is due to aseismic fault motions (*1*–*11*). This phenomenon, known as injection-induced aseismic slip, has been known since at least the 1960s when a slow surface fault rupture was causally linked to fluid injection operations of an oil field in Los Angeles (*1*). Since then, an increasing number of observational studies have inferred the occurrence of slow slip events as a result of industrial fluid injections. For example, in the Brawley Geothermal Field, California, ground- and satellite-based geodetic techniques allowed for the detection of an injection-induced aseismic slip event (*7*, *9*). This event was found to precede and likely trigger a seismic sequence in 2012 (*12*). In western Canada, two of the largest aseismic slip events observed thus far (magnitudes of 5.0 and 4.2) occurred in 2017–2018 and were detected using interferometric synthetic aperture radar (InSAR) measurements of surface deformation (*10*). These events were attributed to hydraulic fractures possibly intersecting glide planes during the stimulation of an unconventional hydrocarbon reservoir (*10*). Similarly, InSAR-derived surface deformations allowed for the recent detection of aseismic ruptures in the southern Delaware Basin, Texas (*11*), likely induced by wastewater injection operations (*13*). These recent geodetic observations, in combination with mounting evidence for aseismic slip from fluid injection field experiments (*2*–*6*, *8*), suggest that injection-induced slow slip events might be a ubiquitous phenomenon, largely underdetected over the past decades only due to the lack of geodetic monitoring.

There is increasing recognition of the importance of injection-induced aseismic slip in the geo-energy industry. For instance, in the development of deep geothermal reservoirs, hydraulic stimulation techniques are commonly used to reactivate preexisting fractures in shear. This process aims to enhance reservoir permeability through the permanent dilation of preexisting fractures or the creation of new ones. The occurrence of predominantly aseismic rather than seismic slip is desirable, as earthquakes of considerable magnitude can pose a substantial hazard to the success of these projects (*14*, *15*). Injection-induced aseismic slip, however, can be detrimental in several ways. For example, aseismic slip on fractures intersecting wells can cause casing shearing (*3*, *10*) and adversely affect well stability (*16*, *17*). In addition, in carbon dioxide (CO_2_) storage operations, injection-induced aseismic slip could affect the integrity of low-permeability caprocks, as fault slip may be accompanied by permeability enhancements, increasing the risk of CO_2_ leakage (*18*, *19*). Similar concerns may arise in other underground operations such as the storage of hydrogen and gas. Furthermore, it is well established that quasi-static stress changes due to aseismic slip may induce seismic failures on nearby unstable fault patches (*2*–*4*, *6*, *7*). Moreover, since aseismic slip can propagate faster than pore pressure diffusion (*6*, *20*, *21*), aseismic slip stress changes can potentially reach regions much further than the zones affected by the direct increase in pore pressure due to injection, thereby increasing the likelihood of triggering earthquakes of undesirably large magnitude by perturbing a larger rock volume (*12*, *21*, *22*).

Understanding the physical factors controlling the spatial extent of aseismic slip is, thus, of great importance to better constrain the influence zone of injection operations concerning seismic hazards. Recent theoretical and numerical modeling studies have provided, within certain simplifying assumptions, a fundamental mechanistic understanding of how injection-induced aseismic slip grows in a realistic three-dimensional context and through all its stages from nucleation to arrest (*23*–*25*). Estimating the rupture run-out distance of aseismic slip transients remains, despite these efforts, an unresolved issue, particularly as these prior investigations have focused only on specific injection protocols (*23*–*25*). However, the spatiotemporal patterns of injection-induced aseismic slip growth are anticipated to be strongly influenced by the history of the injection flow rate (*23*). On the other hand, a related issue is estimating the maximum magnitude of injection-induced earthquakes. This quantity plays a crucial role in earthquake hazard assessment and has been the focus of substantial research efforts in recent times (*26*–*36*). A common limitation of prior research in this area is neglecting the portion of moment release due to aseismic slip, despite considerable evidence suggesting that aseismic motions may contribute importantly to the total moment release (*1*–*11*), potentially surpassing seismic contributions in some cases (*1*–*6*, *8*, *10*, *37*). Understanding the factors governing aseismic moment release is, thus, important and would constitute a first step toward understanding the physical controls on slip partitioning, that is, the relative contributions of aseismic and seismic motions to the release of elastic strain energy, which is crucial for a better understanding of the seismic hazard posed by these operations.

Building upon our previous works (*23*–*25*), we develop here an upper bound model for the temporal evolution of the spatial extent and moment release of injection-induced aseismic slip events that are unconditionally stable. This model is based on a canonical scenario where fluids are injected at a constant volumetric rate up to a specified time, after which the injection is abruptly stopped, capturing both the injection and shut-in stages. Furthermore, using fracture mechanics theory and scaling analysis, we propose scaling relations for the maximum size and magnitude of injection-induced aseismic ruptures, accounting for injection operations with arbitrary flow rate histories during the injection stage, provided the rupture propagates in crack-like mode while injecting. These scaling relations emerge from a rupture regime in which fault slip outpaces pore-fluid migration, representing in situ conditions that yield the largest ruptures for a given injection. Our theoretical predictions are shown to be consistent with a global compilation of events that vary in size from centimeter-scale slip transients monitored in the laboratory to kilometer scale, geodetically inferred slow slip events induced by industrial injections. Furthermore, our results suggest that fault zone storativity (the product between fault zone width and oedometric storage coefficient), background stress change (the background residual shear strength of the fault minus the prestress), and injected fluid volume are crucial quantities in determining the maximum size and magnitude of aseismic ruptures. Notably, the total fluid volume injected by a given operation is shown to be the only operational parameter that matters in determining the maximum size and magnitude of these events, regardless of any other characteristic of the injection protocol.

## RESULTS

### Physical model and upper bound rationale for unconditionally stable ruptures

**Fig. 1.**
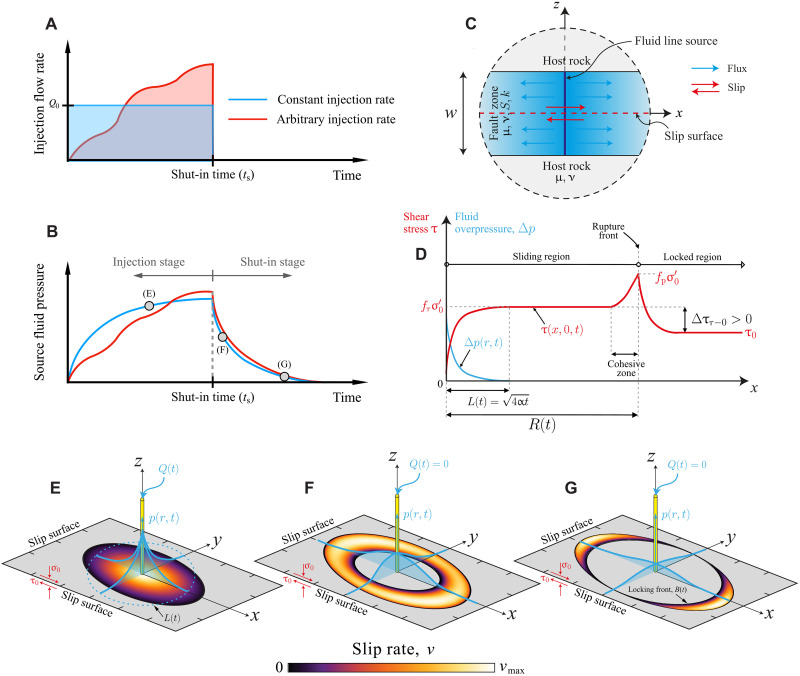
Model schematics. (**A**) Fluid is injected at a constant or arbitrary volumetric rate until the shut-in time *t*_s_ at which the injection is instantaneously stopped. (**B**) This results in two distinct stages: an injection stage and a shut-in stage. (**C**) Details of the porous fault zone near the fluid source. (**D**) Typical shear stress and fluid overpressure distribution along the fault during the injection stage. (**E** to **G**) Distinct stages of rupture propagation in our upper bound model [see (B) indicating the corresponding times as gray circles). (E) Crack-like rupture phase during the injection stage. [(F) and (G)] Pulse-like rupture phase during the shut-in stage: first as a ring-like pulse (F) and after as two crescent-shaped pulses (G). See the main text for a detailed description of the stages.

We consider purely aseismic ruptures nucleated by a localized increase in pore-fluid pressure due to the direct injection of fluids into a porous fault zone of width *w* (see Table 1 for a list of symbols and definitions used throughout this article). Fault slip is assumed to be concentrated in a principal slip zone modeled as a mathematical plane located at *z* = 0 (Fig. 1C). We begin by examining a fluid injection conducted at a constant volumetric rate *Q*_0_ over a finite time *t*_s_, followed by a sudden injection stop (Fig. 1A). This results in two distinct stages: an injection stage in which pore pressure increases everywhere within the permeable fault zone and a shut-in stage in which pore pressure decays near the injection point (Fig. 1B) while transiently increasing away from it (Fig. 1F). Incorporating these two stages into the model allows for examining aseismic ruptures throughout their entire lifetime, from nucleation to arrest. We consider a planar infinite fault obeying a slip-weakening friction law [for example, (*38*)] with a static (peak) friction coefficient *f*_p_, dynamic (residual) friction coefficient *f*_r_, and characteristic slip-weakening distance *d*_c_. The decay of friction from the peak to the residual value can be either linear or exponential (*25*). The host rock is considered purely elastic with the same elastic constants as the fault zone, that is, shear modulus μ and Poisson’s ratio ν (Fig. 1C). We assume the host rock to be impermeable at the relevant timescales of the injection. This configuration is motivated by the permeability structure of fault zones in which a highly permeable damage zone is commonly surrounded by a less permeable host rock (*39*, *40*). Under these assumptions and particularly at large times compared to the characteristic time for diffusion of pore pressure in the direction perpendicular to the fault, *w*^2^/α, with α as the fault zone hydraulic diffusivity, the volumetric deformation rate in the fault zone reduces to its component normal to the fault plane that linearly relates to the pore pressure rate under constant normal stress (*41*). Fluid flow within the fault zone is then governed by an axisymmetric linear diffusion equation for the pore pressure field *p*, $\partial p/\partial t=\alpha \nabla^{2}p$ (*42*), where the hydraulic diffusivity α = *k*/Sη, with *k* and η as the fault permeability and fluid dynamic viscosity, respectively, and *S* as the so-called oedometric storage coefficient (*42*, *43*). By neglecting any poroelastic coupling within the fault zone upon the activation of slip, deformation in the medium is then governed by linear elasticity in its quasi-static approximation due to the slow nature of the slip we are concerned with.

**Table 1.** Symbols, definitions, and units.

Symbol	Definition	Units
#x03B1;	Hydraulic diffusivity of fault zone	m^2^/s
δ(*x*, *y*, *t*)	Distribution of fault slip at time *t*	m
δ_c_(*t*)	Characteristic slip for nearly unstable ruptures	m
ζ(*r*, *t*)	Axisymmetric variation in fluid content within the fault zone at time *t*	–
η	Dynamic viscosity of preexisting and injected fluid	Pa·s
λ(*t*)	Time-dependent amplification factor of the slip-weakening model for constant injection flow rate	–
λ_*r*_	Time-independent amplification factor of the upper bound model for constant injection flow rate defined in Eq. 2	–
λ_*r*_(*t*)	Time-dependent amplification factor of the upper bound model for variable injection flow rate defined in Eq. 28	–
μ	Shear modulus of host rock and fault zone	Pa
ν	Poisson’s ratio of host rock and fault zone	–
σ_0_	Background (in situ) total stress normal to the fault plane	Pa
$\sigma_{0}^{\prime }$	Background (in situ) effective stress normal to the fault plane (= σ_0_ − *p*_0_)	Pa
τ_0_	Background (in situ) fault shear stress or prestress	Pa
Δ*p*(*r*, *t*)	Axisymmetric overpressure (= *p* − *p*_0_) within the fault zone at time *t*	Pa
Δp_*_	Injection intensity for constant injection flow rate defined in Eq. 1	Pa
Δp_*_(*t*)	Time-dependent injection intensity for variable injection flow rate	Pa
Δτ_r−0_	Background stress change defined in Eq. 1	Pa
*d*_c_	Characteristic slip-weakening distance	m
*f*_p_	Peak (or static) friction coefficient	–
*f*_r_	Residual (or dynamic) friction coefficient	–
*k*	Fault zone intrinsic permeability	m^2^
*kw*	Fault zone hydraulic transmissivity	m^3^
*p*_0_	Background (in situ) pore pressure within the fault zone	Pa
*p*(*r*, *t*)	Axisymmetric pore pressure within the fault zone at time *t*	Pa
*t*_a_	Rupture arrest time in the upper bound model	s
*t*_s_	Shut-in time	s
*w*	Fault zone width	m
*wS*	Fault zone storativity	m/Pa
*A*_situ_	Prefactor in Eqs. 3 and 5 associated with in situ conditions	m^–1/2^
*B*(*t*)	Radial position of the locking circular front at time *t*	m
*C*_ν_	Prefactor in Eq. 8 associated with rupture noncircularity	–
*C*_shut-in_	Prefactor in Eq. 8 associated with the shut-in stage	–
*F*(*t*)	Force normal to the fault plane induced by fluid injection at time *t*	N
*I*_situ_	Prefactor in Eqs. 7 and 8 associated with in situ conditions	N·m^–7/2^
*L*(*t*)	Radial position of the nominal overpressure front at time *t*	m
*M*	Prefactor of maximum rupture run-out distance defined in Fig. 6	m^–1/2^
*M*_0_(*t*)	Aseismic moment release at time *t*	N·m
$M_{0}^{\text{max}}$	Maximum aseismic moment release for arbitrary fluid injections and λ_r_ ≫ 1	N·m
$M_{w}^{\text{max}}$	Maximum moment magnitude for arbitrary fluid injections and λ_r_ ≫ 1	–
*N*	Prefactor of maximum moment release defined in Fig. 5	N·m^–7/2^
*P*(*t*)	Radial position of the pore pressure back front at time *t*	m
*Q*_0_	Constant injection flow rate	m^3^/s
*Q*_avg_	Volume-average injection flow rate at the shut-in time	m^3^/s
*Q*_avg_(*t*)	Volume-average injection flow rate at time *t* defined in Eq. 28	m^3^/s
*R*_a_	Arrested rupture radius (or furthest rupture extent along the *x* axis) in the upper bound model	m
*R*_max_	Maximum rupture run-out distance for arbitrary fluid injections and λ_r_ ≫ 1	m
*R*_s_	Radius of circular rupture at the shut-in time	m
*R*(*t*)	Radius of circular rupture at time *t*	m
*S*	Fault zone oedometric storage coefficient	Pa^−1^
*S*_ν_	Prefactor in Eq. 5 associated with rupture noncircularity	–
*S*_shut-in_	Prefactor in Eq. 5 associated with the shut-in stage	–
$T_{r}$	Residual stress–injection parameter for constant injection flow rate defined in Eq. 1	–
$T_{r}\left(t\right)$	Time-dependent residual stress–injection parameter for variable injection flow rate defined in Eq. 28	–
*V*(*t*)	Cumulative injected fluid volume at time *t*	m^3^
*V*_tot_	Total fluid volume injected during an injection operation	m^3^

We presented an investigation of this physical model, which can be regarded as an extension to three dimensions of the two-dimensional plane strain model of Garagash and Germanovich (*44*), in a recent study (*25*). In this model (*25*), slow slip events can occur in two distinct forms, namely, unconditionally stable slip and conditionally stable slip. Conditionally stable slip refers specifically to the nucleation phase of an otherwise dynamic rupture, whereas unconditionally stable slip corresponds to a regime in which ruptures will never transition to a macroscopic dynamic event. As shown by Sáez and Lecampion (*25*), each of these two modes of aseismic slip is characterized by distinct dynamics. In the present article, we focus exclusively on the case of unconditionally stable ruptures [regime R1 in figure 4 of (*25*)]. Unconditionally stable ruptures notably require that the in situ residual fault strength, $f_{r}\sigma_{0}^{\prime }$, exceeds the uniform background shear stress, τ_0_, where $\sigma_{0}^{\prime }=\sigma_{0}-p_{0}$ is the uniform background effective normal stress, with σ_0_ and *p*_0_ as the uniform background total normal stress and pore pressure, respectively. τ_0_ and $\sigma_{0}^{\prime }$ are thought to be the result of long-term tectonic processes and, thus, considered to be constant during the timescales associated with the injection operation.

**Fig. 2.**
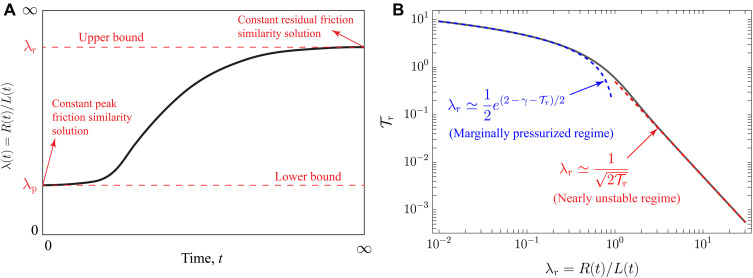
Upper bound rationale and amplification factor. (**A**) Evolution of the amplification factor λ(t)=R(t)/L(t) for unconditionally stable ruptures in the slip-weakening fault model [adapted from figure 9 in (*25*)] for a constant injection flow rate. *R*(*t*) is the rupture radius, and $L\left(t\right)=\sqrt{4\alpha t}$ is the position of the nominal overpressure front. λ_r_ (time independent) is an upper bound for the rupture size at any time during the injection stage. (**B**) Analytical solution (solid black line; see Materials and Methods) for the amplification factor λ_*r*_ in our upper bound model. λ_r_ depends uniquely on the residual stress–injection parameter $T_{r}$. Blue and red dashed lines correspond to asymptotic limiting behaviors for marginally pressurized (λ_r_ ≪ 1) and nearly unstable (λ_r_ ≫ 1) ruptures.

In this model, unconditionally stable ruptures evolve always between two similarity solutions [Fig. 2A and see (*25*) for further details], one at early times where the fault interface operates with a constant friction coefficient equal to the peak value *f*_p_ and the other one at late times where the fault interface behaves as if it were governed by a constant friction coefficient equal to the residual value *f*_>r_. As shown in Fig. 2A, the constant residual friction solution, which is the ultimate asymptotic regime of any unconditionally stable rupture, is an upper bound for the rupture size at any given time during the injection stage. In this asymptotic regime, rupture growth is dictated by a fracture-mechanics energy balance, where the interplay between driving and resisting forces leads to a scenario in which the fracture energy can be effectively neglected (*25*). During the shut-in stage, a similar upper bound rationale can be applied to the case of unconditionally stable ruptures. Assuming the fault slides with a constant residual friction value across the slipping region (equivalent to neglecting the fracture energy in the rupture-tip energy balance), this limiting solution would consistently yield an upper bound for the rupture size, as the effect of the fracture energy is always to slow down rupture advancement. The limiting scenario of constant residual friction serves, therefore, as an effective upper bound model for unconditionally stable ruptures during and after fluid injection. In the following sections, we explore the consequences of such a limiting condition to provide an upper bound for the evolution of the rupture size and moment release from nucleation to arrest. While the canonical example of injection at a constant flow rate is used to comprehensively examine injection-induced aseismic slip across different stages and regimes—ranging from scenarios where the aseismically sliding region remains confined within the pressurized zone to those where it propagates considerably beyond it—we emphasize in advance that our scaling relations for the maximum size and magnitude (representative of cases where the aseismic slip front extends well beyond the pressurized region) will account for injection operations conducted with an arbitrary volumetric rate history (Fig. 1A), provided that during injection the rupture expands in crack-like mode.

### Dynamics of unconditionally stable ruptures and maximum rupture run-out distance

During and after fluid injection, our upper bound model for injection at a constant flow rate *Q*_0_ is governed by a single dimensionless number (see Materials and Methods), the so-called residual stress–injection parameter

$$T_{r}=\frac{\Delta \tau_{r-0}}{f_{r}\Delta p_{*}},\text{with}\;\Delta \tau_{r-0}=f_{r}\sigma \prime_{0}-\tau_{0}\;\text{and}\;\Delta p_{*}=Q_{0}\eta /4\pi \mathrm{kw}$$ (1)

This dimensionless number systematically emerges in physics-based models of injection-induced fault slip (*20*, *23*–*25*, *45*). It quantifies the competition between the two opposite “forces” that determine the dynamics of unconditionally stable ruptures in our upper bound model. One is a driving force associated with the sole effect of pore pressure increase due to fluid injection that continuously reduces fault shear strength, thereby releasing potential energy that becomes available for rupture growth. Its stress scale is $f_{r}\Delta p_{*}$, where $\Delta p_{*}$ is the injection intensity. Injections with faster pressurization are associated with increasing values of Δp_*_, which can occur, for example, because of a higher injection flow rate (*Q*_0_) or a lower hydraulic transmissivity (*kw*). The other force is of a resisting kind, which, in the absence of a local energy dissipation mechanism such as the fracture energy, corresponds to a nonlocal “consumption” of elastic strain energy associated with the background stress change, $\Delta \tau_{r-0}$. The latter, as displayed in Fig. 1D, is defined as the difference between the in situ residual fault strength ($f_{r}\sigma \prime_{0}$) and the initial shear stress (τ_0_). The background stress change is strictly positive, or, in other words, the so-called stress drop is negative. This is an essential feature of unconditionally stable ruptures, which stems from the so-called ultimate stability condition: $\tau_{0}<f_{r}\sigma_{0}^{\prime }$ (*44*). The latter ensures fault stability and the development of quasi-static slip unconditionally in this regime, as ruptures with a positive stress drop will always ultimately transition to a dynamic rupture (*25*). Intuitively, one expects that as the intensity of the injection (Δp_*_) increases, the rupture would propagate faster. Conversely, when the background stress change (Δτ_r−0_) is higher, it presents greater resistance to rupture growth, slowing down the slip propagation. Hence, decreasing $T_{r}$ values will always result in faster aseismic ruptures. This behavior can be observed in Fig. 2B, where the solution (see Materials and Methods) during the injection stage for a circular rupture of radius *R*(*t*) is shown. Here

$$R\left(t\right)=\lambda_{r}L\left(t\right)$$ (2)

where λ_r_ is the so-called amplification factor given by Sáez *et al.* (*23*). Notably, their equation 21 applies here, provided that the stress-injection parameter is understood as the residual one, $T_{r}$. $L\left(t\right)=\sqrt{4\alpha t}$ is the classical diffusion length scale, also considered as the position of the overpressure front (Fig. 1E). Two important points must be emphasized. First, the definition of the overpressure front *L*(*t*) is only nominal. Strictly speaking, even immediately after injection begins, the overpressure in our model only approaches zero at infinity. More precisely, the overpressure front *L*(*t*) tracks an isobar of fluid pressure, typically in the order of 1% of the pressure at the fluid source at any given time. This definition is exact only when the overpressure distribution is self-similar; in all other cases, it should be considered an approximation. Nonetheless, *L*(*t*) is a proxy for the boundaries of the pressurized zone resulting from injection. Second, the analytical circular rupture solution for λ_r_ as a function of $T_{r}$ (Fig. 2B) is strictly valid only when Poisson’s ratio ν = 0 (*23*). This is due to the axisymmetry property of the fluid flow problem, which, together with the condition ν = 0, results in an energy release rate that is uniform along the rupture front (*23*). Throughout this work, we generally adopt the circular rupture approximation to derive purely analytical insights. We, nevertheless, quantify the effect of rupture noncircularity numerically via a boundary element–based numerical solver (see Materials and Methods).

The analytical solution in Fig. 2B provides important insights into the response of our upper bound model. During the injection stage, the fault response is characterized by two distinct regimes. When $T_{r}∼10$, aseismic ruptures are confined well within the overpressurized region (λ_r_ ≪ 1), a regime known as marginally pressurized because it relates to a scenario in which the fluid injection provides just the minimum amount of overpressure that is necessary to activate fault slip (*25*, *44*). Conversely, when $T_{r}\ll 1$, aseismic ruptures break regions much further away than the pressurized fault zone (λ_r_ ≫ 1). This is the so-called nearly unstable regime (*25*) as when $\Delta \tau_{r-0}\to 0$, the fault approaches the so-called ultimate stability condition after which any positive stress drop will ultimately lead to a frictional instability.

From a practical standpoint, the nearly unstable regime is the most relevant one to derive scaling relations for the maximum rupture size and moment release, as it produces the largest ruptures for a given injection. While operators in geo-energy applications typically maintain good control over fluid injection parameters, in situ conditions, such as frictional parameters and the stress state acting upon fractures and faults within a reservoir, are subject to considerable uncertainties. Given that in situ conditions largely control the response of aseismic slip in our model, it is conservative to assume, under generally uncertain and somewhat generic conditions in the rock mass surrounding a given operation, that the nearly unstable regime develops to establish the desired upper limits. Consequently, our calculations for the maximum rupture size *R*_max_ and moment release $M_{0}^{\text{max}}$ will be based on this regime. When λ_r_ ≫ 1, one can derive a relation linking the evolution of the rupture radius to the accumulated injected fluid volume [*V*(*t*)] and in situ conditions as follows (see Materials and Methods)

$$R\left(t\right)=A_{\text{situ}}\sqrt{V\left(t\right)},\text{with}\;A_{\text{situ}}={\left(\frac{f_{r}}{2\pi \text{wS}\Delta \tau_{r-0}}\right)}^{1/2}$$ (3)

Furthermore, we demonstrate (see Materials and Methods) that Eq. 3 is valid not only for injection at a constant flow rate but also for any arbitrary fluid injection as long as the rupture propagates in crack-like mode while injecting. Crack-like behavior is a fundamental requirement in our model, as this latter assumes that the shear stress distribution across the circular sliding region, 0 ≤ *r* ≤ *R*(*t*), equals the fault shear strength at any time during the injection stage (*t* ≤ *t*_s_). An abrupt drop in fluid pressure, such as that caused by halting injection, can trigger propagation in a pulse-like mode (*24*), creating a region within the reactivated fault surface where the fault strength locally exceeds the shear stress. While the precise conditions of the fluid source necessary to ensure crack-like propagation remain unknown, this rupture propagation mode is guaranteed when the overpressure from fluid injection increases monotonically across 0 ≤ *r* ≤ *R*(*t*). A specific example is when overpressure remains constant at the fluid source, leading to a sharp rise in injection flow rate, followed by a gradual decline over time (*23*). This example demonstrates that crack-like behavior can occur even under conditions of decreasing flow rate.

Equation 3 implies that as long as the aseismic slip front substantially outpaces the overpressure front (λ_r_ ≫ 1), the cumulative injected fluid volume *V*(*t*) is the only operational parameter of the injection that matters to obtain an upper bound for the rupture size during the injection. In practice, asymptotic expressions associated with the nearly unstable regime, such as Eq. 3, are expected to provide good approximations even when λ_r_ is close to one. For instance, the analytical solution for constant flow rate injection shown in Fig. 2B indicates that the nearly unstable asymptote approximates the exact solution with a relative error of only 8% at λ_r_ = 1. Furthermore, the prefactor in Eq. 3 is exclusively related to in situ conditions (*A*_situ_), highlighting a clear separation between contributions to rupture size that are controllable during an operation [*V*(*t*)] and those that are not (*A*_situ_). This distinction is advantageous for geo-energy operators, as it directly relates the controllable parameter [*V*(*t*)] to the maximum rupture run-out distance from injection wells. This distance, in turn, provides a practical estimate of the area potentially affected by aseismic slip–induced stress changes and the subsequent triggering of earthquakes. Note that along the fault, seismicity can be triggered beyond the slip front due to stress amplification ahead of the rupture (*21*). In addition, seismicity may occur at greater distances off-fault, such as in regions experiencing positive Coulomb stress changes on adjacent faults (*24*, *37*). Therefore, the rupture run-out distance should be understood as an order-of-magnitude estimate of the region where earthquake triggering might occur.

The in situ factor *A*_situ_ reflects clear physical controls on rupture size. Equation 3 indicates that a decrease in the background stress change (Δτ_r−0_) results in an increase in the rupture radius. The reason behind this behavior is simple, lower background stress variations reduce the resistance to rupture growth, allowing for larger ruptures. For the same reason, higher values of the residual friction coefficient (*f*_r_) also augment *R*. On the other hand, decreasing the product between the fault zone width and oedometric storage coefficient (*wS*), namely, the fault zone storativity, also leads to larger ruptures. In this case, the explanation is that *wS* controls the pressurization intensity due to fluid injection that is experienced on average over the fault-pressurized region. A lower storativity in the fault zone naturally implies a higher fluid pressure to accommodate a fixed amount of injected volume (see Eq. 20 in the Materials and Methods). A higher fluid overpressure decreases fault shear strength, therefore increasing the mechanical energy available for rupture growth and the corresponding rupture size.

**Fig. 3.**
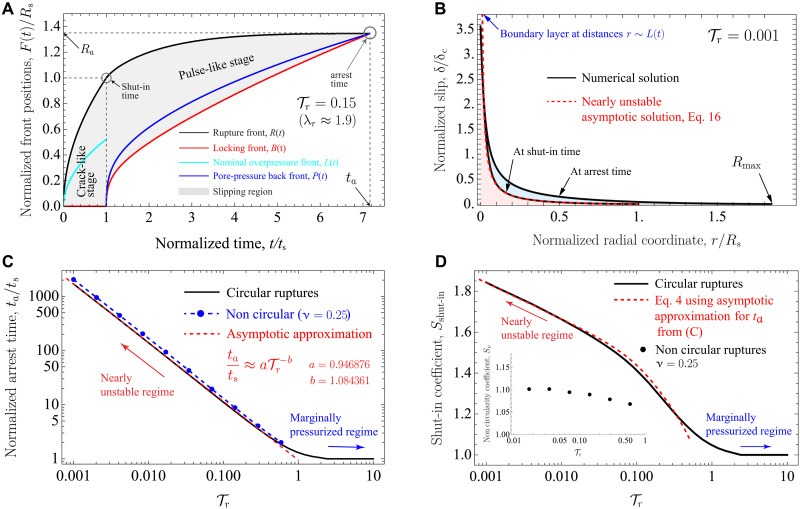
Upper bound model for unconditionally stable ruptures: dynamics, arrest time, and maximum rupture run-out distance. (**A**) Evolution of fluid- and slip-related fronts, during the entire lifetime of an injection-induced aseismic slip event, for a case with $T_{r}=0.15$. Front positions *F*(*t*) = *R*(*t*), *B*(*t*), *L*(*t*), and *P*(*t*) are normalized by the rupture radius at the shut-in time, *R*_s_ (see main text for a description of the different fronts). (**B**) Slip distribution for a very nearly unstable rupture ($T_{r}\ll 1$) at the shut-in and arrest times. $\delta_{c}\left(t\right)=f_{r}\Delta p_{*}L\left(t\right)/\mu$ is the characteristic slip scale in this regime (see Materials and Methods). Slip further accumulates during the shut-in stage due to the slip pulse that travels along the fault upon the shut-in of the injection. (**C**) Upper bound for the arrest time and (**D**) maximum rupture run-out distance as a function of the dimensionless parameter $T_{r}$. In (D), the main plot quantifies the shut-in coefficient *S*_shut-in_ = *R*(*t*_a_)/*R*_s_ for a circular rupture. Nearly unstable ruptures can grow up to ≈2 times their size at the shut-in time. Conversely, marginally pressurized ruptures ($T_{r}∼10$) experience no further growth during the shut-in stage. In the inset, the noncircularity coefficient *S*_ν_ as a function of $T_{r}$ for ν = 0.25. *S*_ν_ shows that over a wide range of practically relevant cases ($0.01\leq T_{r}\leq 1$), the maximum rupture run-out distance of a noncircular rupture is about 6 to 10% larger than the one of a circular rupture at same $T_{r}$.

Upon the stop of the fluid injection (*t* > *t*_s_), our upper bound model produces ruptures that transition from crack-like to pulse-like propagation mode (Fig. 1, E to G). Since we reduced the upper bound problem to a fault responding with a constant friction coefficient equal to the residual value *f*_r_, we inherit essentially all the results obtained recently by Sáez and Lecampion (*24*) who extensively investigated the propagation and arrest of postinjection aseismic slip on a fault with constant friction. In particular, the overpressure drops quickly near the fluid source upon stopping the injection, while it keeps increasing transiently away from it (Fig. 1, E and F). This latter increase in pore pressure further drives the propagation of aseismic ruptures after shut-in. As shown in Fig. 1F, slip propagates first as a ring-shaped pulse with a locking front that always propagates faster than the rupture front (Fig. 3A). The locking front is driven by the continuous depressurization of pore fluids, which restrengthens the fault. After and for the more general case of noncircular ruptures, the pulse splits into two “crescent-shaped” pulses (Fig. 1G). This ultimate stage is due to the locking front catching up with the rupture front first in the less elongated side of the slipping region. For the idealized case of circular ruptures, the crescent-shaped pulses are absent because of the axisymmetry property of both the fluid flow and shear rupture problems. Figure 3A displays the evolution of the locking front *B*(*t*) and rupture front *R*(*t*) for a circular rupture for an exemplifying case with $T_{r}=0.15$. Slip arrests when the locking front catches the rupture front at the time *t*_a_ (arrest time or duration of the slow slip event), resulting in the arrested rupture radius *R*_a_. For the more general case of noncircular ruptures, the furthest rupture run-out distance from the injection well *R*_a_ occurs always along the *x* axis, which is the direction of maximum shear before the injection starts (Fig. 1G). Moreover, the rupture front stops when it is caught by the so-called pore pressure back front *P*(*t*) (*24*) introduced by Parotidis *et al.* (*46*). The pore pressure back front tracks the radial distance from the injection well at which the pore pressure rate is zero at a given time *t*, that is, ∂*p*/∂*t* = 0 at *r* = *P*(*t*). Then, the region in which overpressure is decreasing is *r* < *P*(*t*), and, conversely, the overpressure is increasing at *r* > *P*(*t*). Hence, the moment when the pore pressure back front catches up with the rupture front (*t*_a_) marks the point at which no additional increase in pore pressure within the rupture pulse is available to sustain any further quasi-static slip propagation: a necessary condition for rupture arrest (*24*). Furthermore, this arrest condition leads to the following analytical relation between the rupture run-out distance *R*_a_ and the arrest time *t*_a_

$$R_{a}={\left[4\alpha t_{a}\left(\frac{t_{a}}{t_{s}}-1\right)\text{ln}\left(\frac{t_{a}}{t_{a}-t_{s}}\right)\right]}^{1/2}$$ (4)

The arrest condition underpinning the previous equation is, in principle, valid for any arbitrary fluid injection. Its mathematical form (Eq. 4) is, however, particular to the case of injection at a constant volume rate (*24*). In the nearly unstable regime ($T_{r}\ll 1$) and for the particular case of circular ruptures, the normalized arrest time (Fig. 3C) can be estimated via the following numerically derived asymptotic approximation, $t_{a}/t_{s}\approx aT_{r}^{-b}$, with *a* = 0.946876 and *b* = 1.084361. Moreover, Fig. 3C shows that when ruptures are marginally pressurized ($T_{r}∼10$), the slip pulses arrest almost immediately after the injection stops. Conversely, when ruptures are nearly unstable ($T_{r}\ll 1$), the upper bound for the arrest time (*t*_a_) is predicted to be several orders of magnitude greater than the injection duration (*t*_s_). Rupture noncircularity has the effect of slightly increasing the arrest time (Fig. 3C).

To quantify the effect of the shut-in stage in the maximum rupture run-out distance *R*_max_ when λ_r_ ≫ 1, we introduce the shut-in coefficient *S*_shut-in_ equal to the ratio between the rupture radius at the time in which a circular rupture arrest, *R*_a_, and the rupture radius at the shut-in time, *R*_s_. By dimensional analysis, the shut-in coefficient depends only on the residual stress–injection parameter $T_{r}$, whose relation is calculated numerically and shown in Fig. 3D. The contribution of the shut-in stage to *R*_max_ is approximately a factor of 2 at most when ruptures are very nearly unstable ($T_{r}∼0.001$). Similarly, we quantify the effect of rupture noncircularity by introducing the coefficient *S*_ν_ equal to the ratio between the maximum rupture run-out distance for noncircular ruptures (ν ≠ 0) and the same quantity for the circular case (ν = 0). Again, by dimensional considerations, *S*_ν_ depends only on $T_{r}$ for a given ν. This is shown in the inset of Fig. 3D for the particular case of a Poisson’s solid (ν = 0.25, a common approximation for rocks). The effect of Poisson’s ratio is to increase the maximum run-out distance of a noncircular rupture by about 6 to 10% with respect to a circular one, for the same $T_{r}$. We find this to be valid over a wide range of practically relevant cases ($0.01\leq T_{r}\leq 1$). We conclude that the order of magnitude of *R*_max_ comes directly from evaluating *R*(*t*) in the analytical solution displayed in Fig. 2B at the shut-in time, which, in the regime λ_r_ ≫ 1, takes a more insightful expression given by Eq. 3 that is valid for arbitrary fluid injections provided the rupture propagates in crack-like mode during the injection stage. Using this latter expression, we can calculate the maximum run-out distance when λ_r_ ≫ 1 as

$$R_{\text{max}}=S_{\nu }S_{\text{shut}-\text{in}}A_{\text{situ}}\sqrt{V_{\text{tot}}}$$ (5)

where *V*_tot_ = *V*(*t*_s_) is the total volume of fluid injected during a given operation.

Equation 5 has a multiplicative form, thus effectively factorizing contributions from the injected fluid volume, in situ conditions, shut-in stage, and rupture noncircularity to the maximum rupture run-out distance. Note that both *S*_shut-in_ and *S*_ν_ depend on $T_{r}$ and, thus, also on in situ conditions and parameters of the injection (Eq. 1). However, the effect of the in situ conditions and injection protocol are for the most part contained in *A*_situ_ and *V*_tot_, respectively, which can vary over several orders of magnitude. On the contrary, the dimensionless coefficients *S*_shut-in_ and *S*_ν_ remain always of order one. *S*_shut-in_ and *S*_ν_ are, strictly speaking, defined for constant injection flow rates. However, for other injection protocols, these coefficients could be approximately estimated using, for instance, the volume-average injection flow rate, $Q_{\text{avg}}=\left(1/t_{s}\right)\int_{0}^{t_{s}}Q\left(t\right)dt$. The previous approximation guarantees that the same amount of fluid volume is injected over the same injection period *t*_s_ by both the equivalent constant-rate source *Q*_avg_ and the time-varying arbitrary source *Q*(*t*).

### Maximum moment release and magnitude

**Fig. 4.**
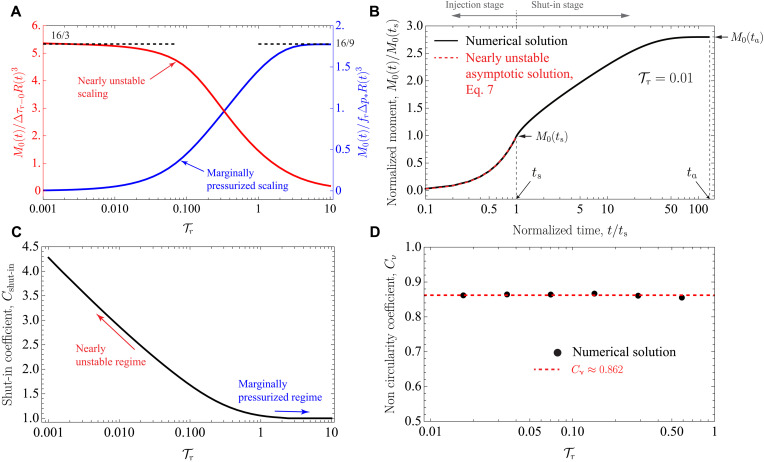
Upper bound model for the moment release from nucleation to arrest. (**A**) Normalized moment release during the injection stage as a function of the residual stress–injection parameter $T_{r}$ using the nearly unstable (red; left axis) and marginally pressurized (blue; right axis) scalings. Black dashed lines correspond to asymptotic analytical solutions provided in the main text. Solid lines correspond to numerical solutions. (**B**) Evolution of the moment release for a nearly unstable circular rupture ($T_{r}\ll 1$) during and after fluid injection. The moment release increases up to ≈3 times the moment release at the shut-in time in this particular case. (**C**) Shut-in coefficient *C*_shut-in_ = *M*_0_(*t*_a_)/*M*_0_(*t*_s_) for a circular rupture as a function of the dimensionless parameter $T_{r}$. Nearly unstable ruptures can experience a moment release increment of up to ≈4 times during the shut-in stage. Conversely, marginally pressurized ruptures ($T_{r}∼10$) are characterized by no increment at all. (**D**) Noncircularity coefficient *C*_ν_ as a function of $T_{r}$ for ν = 0.25. The numerical simulations suggest that over a wide range of practically relevant cases ($0.01\leq T_{r}\leq 1$), the moment release of a noncircular rupture is about 13.8% smaller than the moment release of a circular rupture (ν = 0) at same $T_{r}$.

To calculate the moment release, we derive analytical upper bounds for the spatiotemporal evolution of fault slip during the injection stage, for both nearly unstable (λ_r_ ≫ 1) and marginally pressurized (λ_r_ ≪ 1) circular ruptures (see Materials and Methods). Notably, the slip distribution of nearly unstable ruptures is highly concentrated around the injection point due to an interior boundary layer associated with the fluid injection force at distances *r* ∼ *L*(*t*) (Fig. 3B). Upon integrating the analytical slip distributions over the rupture surface, the temporal evolution of moment release for a circular rupture is

$$M_{0}≃\left\{\begin{array}{c} \frac{16}{3}\Delta \tau_{r-0}R^{3}\;\text{for nearly unstable ruptures},\;\lambda_{r}\gg 1\\ \frac{16}{9}f_{r}\Delta p_{*}R^{3}\;\text{for marginally pressurized ruptures},\lambda_{r}\ll 1 \end{array}\right.$$ (6)

with the temporal dependence of *M*_0_ embedded implicitly in *R*(*t*) = λ_r_*L*(*t*) (Eq. 2), which is known analytically (Fig. 2B). As expected, the previous asymptotic solutions for *M*_0_ match very closely the full numerical solution (Fig. 4A). The numerical solution helps us to describe the precise transition between the two end members. We emphasize that the structure of the scaling for *M*_0_ is the one expected for a circular crack (*M*_0_ ∝ *R*^3^). However, the prefactors and relevant stress scales are specific to the characteristic loading of each regime. For instance, in the nearly unstable regime (λ_r_ ≫ 1), the proper stress scale is the background stress change (Δτ_r−0_), as opposed to the injection intensity (*f*_r_Δp_*_), which is the adequate stress scale when λ_r_ ≪ 1. This is because, in the nearly unstable regime, most of the slipping region experiences a uniform stress variation Δτ_r−0_ except for a small region of size ∼*L*(*t*) near the fluid source, which undergoes an additional nonuniform stress change due to the fluid injection (Fig. 1D). The effect of the fluid injection force is in the prefactor 16/3, which is about two times bigger than the one of a circular crack with purely uniform stress drop [16/7 when ν = 0.25 (*47*) and 8/3 when ν = 0 (*48*)]. Moreover, in the nearly unstable regime, we obtain the following expression for the moment release, which is valid for arbitrary fluid injections as long as the rupture propagates in crack-like mode during injection (see Materials and Methods)

$$M_{0}\left(t\right)=I_{\text{situ}}\cdot V{\left(t\right)}^{3/2},\text{with}\;I_{\text{situ}}=\frac{16}{3{\left(2\pi \right)}^{3/2}}\frac{1}{\sqrt{\Delta \tau_{r-0}}}{\left(\frac{f_{r}}{\mathrm{wS}}\right)}^{3/2}$$ (7)

Equation 7 has the same property as Eq. 3, that is, the only operational parameter of the injection controlling the upper bound for the moment release in the nearly unstable regime and during the injection stage is the cumulative injected fluid volume *V*(*t*). Furthermore, the prefactor corresponds as well to in situ conditions (*I*_situ_), thus separating contributions to the moment release that are controllable during an operation [*V*(*t*)] from those that are not (*I*_situ_). This kind of relation for the moment release has been previously reported in the literature for the case of regular, fast earthquakes (*27*, *28*, *30*, *32*, *35*). In the marginally pressurized regime (λ_r_ ≪ 1), *M*_0_ does not follow an expression as in Eq. 7. By substituting the expressions $\Delta p_{*}=Q_{0}\eta /4\pi \text{kw}$, $R\left(t\right)=\lambda_{r}\sqrt{4\alpha t}$, and *V*(*t*) = *Q*_0_*t* into Eq. 6, one can readily show that the moment release for an injection at a constant flow rate is given by $M_{0}\left(t\right)=B\cdot V{\left(t\right)}^{3/2}$, with $B=\left(32/9\pi \right)\left(f_{r}\eta /\mathrm{kw}\right)\left(\lambda_{r}^{3}\alpha^{3/2}/Q_{0}^{1/2}\right)$. This implies that in the marginally pressurized regime, the moment release depends on both the current injected volume *V*(*t*) (or injection time *t*) and the injection rate *Q*_0_ (which is also implicitly in λ_r_). The in situ and operational factors cannot be separated as in Eq. 7. This separation is a unique characteristic of nearly unstable ruptures, associated with the fact that when λ_r_ ≫ 1, the effect of the fluid source on the propagation of the rupture front is entirely described by an equivalent point force at distances *r* ≫ *L*(*t*). The magnitude of this point force is determined uniquely by the injected fluid volume, irrespective of any other details of the fluid injection (see Eq. 23 in Materials and Methods).

The influence of in situ conditions (*I*_situ_) on moment release *M*_0_ is almost identical to its role in controlling rupture size. Equation 7 reflects the fact that a decrease in background stress change (Δτ_r−0_) represents less opposition for the rupture to growth, thus leading to an increase in moment release (and the same is true for a decrease in the residual friction coefficient *f*_r_). On the other hand, a decrease in fault zone storativity (*wS*) also leads to a larger moment release, as it implies a stronger pressurization for a fixed amount of injected fluid volume.

During the shut-in stage (*t* > *t*_s_), the propagation and ultimate arrest of the aseismic slip pulses result in a further accumulation of fault slip (Fig. 3B). The shut-in stage, thus, increases the final, maximum moment release of the events. Figure 4B displays the evolution of this increase for an exemplifying case with $T_{r}=0.01$. We observe that the moment release keeps growing after shut-in very slowly (over a timescale that is about 100 times the injection duration) up to reaching (at arrest) nearly three times the moment release at the time the injection stops [*M*_0_(*t*_s_)]. We quantify this effect in the same manner as for the maximum rupture run-out distance *R*_max_, by defining the shut-in coefficient *C*_shut-in_ equal to the ratio between the moment release at the time in which a circular rupture arrest, *M*_0_(*t*_a_), and the moment release at the shut-in time, *M*_0_(*t*_s_). By dimensional analysis, the shut-in coefficient depends only on the residual stress–injection parameter $T_{r}$, whose relation is calculated numerically and displayed in Fig. 4C. We observe that *M*_0_(*t*_a_) is at most around four times the moment release at the time the injection stops in the more nearly unstable cases (smallest values of $T_{r}$). Conversely, there is virtually no further accumulation of moment release for marginally pressurized ruptures. Similarly, we quantify the effect of rupture noncircularity by introducing the coefficient *C*_ν_ equal to the ratio between the moment release at the time of arrest for noncircular ruptures (ν ≠ 0) and the same quantity for the circular case (ν = 0). Again, by dimensional considerations, *C*_ν_ depends only on $T_{r}$ for a given ν. This is shown in Fig. 4D for the particular case of a Poisson’s solid (ν = 0.25). We observe that over a wide range of practically relevant cases ($0.01\leq T_{r}\leq 1$), the effect of Poisson’s ratio is to reduce the moment release of a noncircular rupture by about 13.8% with respect to that of a circular one, for the same $T_{r}$.

With all the previous definitions and calculations, we can lastly estimate the maximum moment release associated with the nearly unstable regime (λ_r_ ≫ 1) as $M_{0}^{\text{max}}=C_{\nu }\cdot C_{\text{shut}-\text{in}}\cdot M_{0}\left(t_{s}\right)$. Equation 7, which is valid for arbitrary fluid injections, can then be evaluated at the shut-in time, leading to the following expression

$$M_{0}^{\text{max}}=C_{\nu }\cdot C_{\text{shut}-\text{in}}\cdot I_{\text{situ}}\cdot V_{\text{tot}}^{3/2}$$ (8)

where *V*_tot_ is the total volume of fluid injected during a given operation. As in Eq. 5, Eq. 8 has a multiplicative structure, thus factorizing contributions from the injected fluid volume, in situ conditions, shut-in stage, and rupture noncircularity to the maximum moment release. Here, *C*_shut-in_ and *C*_ν_ depend also on $T_{r}$ and, thus, on in situ conditions and injection parameters (Eq. 1). However, similar to the case of *R*_max_, since the dimensionless coefficients *C*_shut-in_ and *C*_ν_ always remain of order one, the order of magnitude of $M_{0}^{\text{max}}$ is controlled essentially by the in situ factor *I*_situ_ and injected volume *V*_tot_. Moreover, since *C*_shut-in_ and *C*_ν_ are strictly defined for constant flow rate injections, their values for variable injection flow rates *Q*(*t*) could be again approximated using, for example, the volume-average injection rate, $Q_{\text{avg}}=\left(1/t_{s}\right)\int_{0}^{t_{s}}Q\left(t\right)dt$.

To calculate the maximum magnitude, we follow the definition by Hanks and Kanamori (*49*): $M_{w}^{\text{max}}=2/3\cdot \left[{\text{log}}_{10}\left(M_{0}^{\text{max}}\right)-9.1\right]$ (here, in the International System of Units). Substituting Eq. 8 into the previous expression leads to the following estimate for the maximum magnitude

$$M_{w}^{\text{max}}={\text{log}}_{10}\left(V_{\text{tot}}\right)+\frac{2}{3}\left[{\text{log}}_{10}\left(I_{\text{situ}}\right)+{\text{log}}_{10}\left(C_{\text{shut}-\text{in}}\right)+{\text{log}}_{10}\left(C_{\nu }\right)-9.1\right]$$ (9)

Because of the multiplicative form of Eq. 8, Eq. 9 takes an additive form that separates contributions from different factors to the maximum magnitude of injection-induced slow slip events. Among these factors, rupture noncircularity decreases the magnitude by only 0.06. The contribution from the shut-in stage is, on the other hand, slightly larger. Since *C*_shut-in_ ≈ 4 at most when ruptures are very nearly unstable ($T_{r}∼0.001$), the shut-in stage may contribute to an increase in the moment magnitude of 0.4 at the maximum. The larger contributions to $M_{w}^{\text{max}}$ are by far the ones associated with in situ conditions and the total injected fluid volume. For example, a 10-fold increase in *V*_tot_ gives a magnitude increase of 1.0, while a 10-fold increase in *I*_situ_ results in a magnitude growth of approximately 0.67. The relative contributions from the subfactors composing *I*_situ_ can be further understood by substituting Eq. 7 into Eq. 9 and then isolating the in situ term as follows

$$\left(2/3\right){\text{log}}_{10}\left(I_{\text{situ}}\right)={\text{log}}_{10}\left(f_{r}\right)-\left(1/3\right){\text{log}}_{10}\left(\Delta \tau_{r-0}\right)-{\text{log}}_{10}\left(\mathrm{wS}\right)-0.3135$$ (10)

The more substantial variations in $M_{w}^{\text{max}}$ come clearly from the fault zone storativity (*wS*) and background stress change (Δτ_r−0_), which could vary over several orders of magnitude. For instance, a variation of three orders of magnitude in Δτ_r−0_ yields a change of magnitude of 1.0, while the same variation in fault zone storativity results in a magnitude change of 3.0, highlighting the potentially strong effect of *wS* in $M_{w}^{\text{max}}$.

### Fault zone storativity, background stress change, and injected fluid volume: Three key parameters

**Fig. 5.**
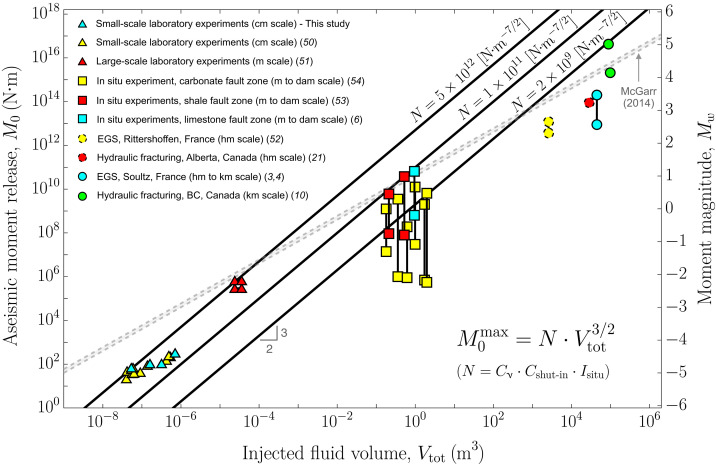
Comparison of our scaling relation for the maximum magnitude $M_{w}^{\text{max}}$ with estimates of moment magnitude from injection-induced slow slip events, as a function of the total injected fluid volume. We consider three values of the factor $N=C_{\nu }\cdot C_{\text{shut}-\text{in}}\cdot I_{\text{situ}}$ (solid black lines), which collectively form an upper limit for the data across different volume and moment release scales. The factor *N* encapsulates the effects of in situ conditions (*I*_situ_), the shut-in stage (*C*_shut-in_), and rupture noncircularity (*C*_ν_). For some events in the dataset, the moment release is estimated within a range that is represented by a vertical line connecting their maximum and minimum values. In addition, the data points shown with a dashed perimeter (*21*, *52*) have considerably greater uncertainty in their moment release compared to the rest of the dataset (see Supplementary Text). Gray dashed lines represent McGarr’s relation (*28*) for shear moduli of 20 and 30 GPa.

**Fig. 6.**
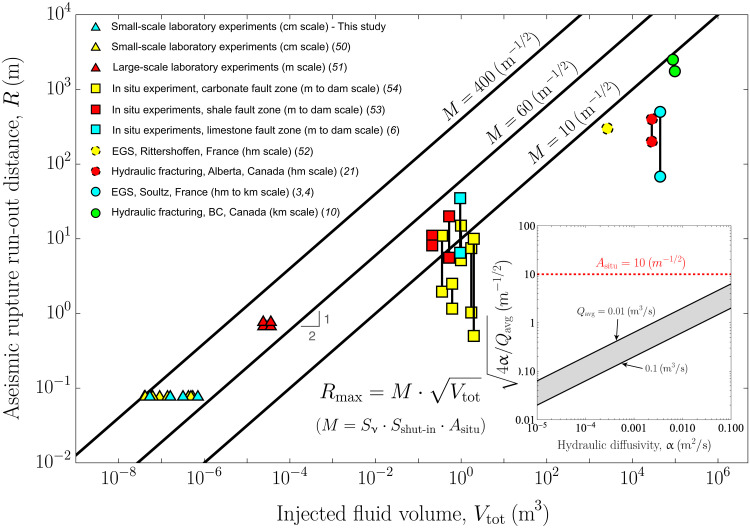
Comparison of our scaling relation for the maximum rupture run-out distance *R*_max_ with estimates of rupture extent from injection-induced slow slip events, as a function of the total injected fluid volume. The events in this figure are the same as in Fig. 5. We consider three values of the factor $M=S_{\nu }\cdot S_{\text{shut}-\text{in}}\cdot A_{\text{situ}}$ (solid black lines), which, together, form an upper limit for the data across different volume and rupture run-out distance scales. The factor *M* encapsulates the effects of in situ conditions (*A*_situ_), the shut-in stage (*S*_shut-in_), and rupture noncircularity (*S*_ν_). Likewise in Fig. 5, for some events, the rupture extent is estimated within a range that is represented by a vertical line connecting their maximum and minimum values. In addition, the data points shown with a dashed perimeter (*21*, *52*) have considerably greater uncertainty in their rupture extent compared to the rest of the dataset (see Supplementary Text). In the inset, the quantity $\sqrt{4\alpha /Q_{\text{avg}}}$ is plotted as a function of fault zone hydraulic diffusivity α across a range of injection flow rates typical of industrial operations (represented by the gray region between the two solid black lines). An in situ factor *A*_situ_ of 10 m^–1/2^ (dashed red line), representative of the upper limit for industrial fluid injections in the main plot, satisfies the condition $A_{\text{situ}}\gg \sqrt{4\alpha /Q_{\text{avg}}}$ over a wide range of practically relevant scenarios. This demonstrates (see main text for a detailed discussion) that the upper limit is consistent with the nearly unstable regime (λ_r_ ≫ 1) underlying the scaling relations for *R*_max_ and $M_{w}^{\text{max}}$.

To test our scaling relations for the maximum rupture run-out distance *R*_max_ and moment release $M_{0}^{\text{max}}$, we compiled and produced a dataset (see Supplementary Text) with estimates of aseismic moment release, rupture run-out distance, and injected fluid volumes from events that vary in size from laboratory experiments (centimeter- to meter-scale ruptures) (*50*, *51*) to industrial applications (hectometer- to kilometer-scale ruptures) (*3*, *4*, *10*, *21*, *52*), including in situ experiments in shallow natural faults at intermediate scales (meter- to decameter-scale ruptures) (*6*, *53*, *54*). The comparison between this dataset and our expressions for the maximum moment release (Eq. 8) [or magnitude (Eq. 9)] and maximum rupture run-out distance (Eq. 4) is displayed in Figs. 5 and 6, respectively. We focus first on the maximum moment release (Fig. 5). To facilitate the comparison against the dataset, we introduce in Fig. 5 the factor $N=C_{\nu }\cdot C_{\text{shut}-\text{in}}\cdot I_{\text{situ}}$, which encapsulates all effects other than the injected fluid volume, so that Eq. 8 can be written simply as $M_{0}^{\text{max}}=N\cdot V_{\text{tot}}^{3/2}$. Three different values for *N* are considered in Fig. 5, which collectively form an upper bound for the data across the different volume and moment release scales characterizing the dataset. Considering that *C*_ν_ ≈ 0.862 and that plausible values for the coefficient *C*_shut-in_ range from 1 to 4, the order of magnitude and units of *N* are determined by the in situ factor (*I*_situ_). This latter, in turn, depends on three parameters: the residual friction coefficient *f*_r_ (with a plausible range of 0.4 to 0.8), the background stress change (Δτ_r−0_), and the fault zone storativity (*wS*). The background stress change can be at most equal to the amount of shear stress that is necessary to activate fault slip before the injection starts, $\Delta \tau_{p-0}=f_{p}\sigma \prime_{0}-\tau_{0}$, in the limiting case in which the weakening of friction is small (*f*_r_ ≈ *f*_p_). Its minimum value could be, on the other hand, as small (but positive) as possible when the residual fault strength drops close to the initial shear stress ($f_{r}\sigma_{0}^{\prime }\approx \tau_{0}$). This is, as already discussed, the case that would promote larger ruptures and moment release. $\Delta \tau_{r-0}$ could therefore reasonably fluctuate between some megapascals and a few kilopascals. The fault zone storativity (*wS*), which is the product between the fault zone width (*w*) and the oedometric storage coefficient (*S*), can similarly span several or even many orders of magnitude. This variability arises from the wide range of values observed in fault zone width (*55*, *56*) and the storativity characteristics of rocks, fractures, and faults (*57*–*60*). Estimating *wS* is quite challenging; however, as anticipated by Eq. 10, the fault zone storativity could have a strong effect on the maximum magnitude. Hence, we conduct a more intricate analysis of representative values for *wS* within our compilation of events.

To do so, we examine the end members of our dataset, namely, small-scale laboratory experiments and industrial-scale fluid injections. Let us first note that in our model, *wS* can be written in terms of generally more accessible quantities as *kw*/αη, where *kw* is the fault zone hydraulic transmissivity, η is the fluid dynamic viscosity, and α is the fault zone hydraulic diffusivity. At the centimeter scale composing the smallest aseismic slip events in the dataset, Passelègue *et al.* (*50*) estimated the hydraulic transmissivity of their saw-cut granitic fault within 10^−17^ and 2 × 10^−18^ m^3^ and its hydraulic diffusivity from 3 × 10^−5^ to 10^−6^ m^2^/s (*61*), at confining pressures ranging from 20 to 100 MPa, respectively. Considering a water dynamic viscosity at the room temperature the experiments were conducted, η ∼ 10^−3^ Pa · s, we estimate *wS* to be within 3 × 10^−10^ and 2 × 10^−9^ m/Pa (assuming that *kw* and α are positively correlated). Taking into consideration the aforementioned characteristic range of values for *f*_r_ and Δτ_r−0_, we estimate the maximum value for the in situ factor that is representative of these laboratory experiments to be roughly *I*_situ_ ∼ 10^12^ N · m^−7/2^. The upper bound for the moment release resulting from this value of *I*_situ_ aligns closely with our estimates of moment release and injected fluid volumes for this very same set of experiments (Fig. 5, yellow triangles). Note that in Fig. 5, the factor *N* must always be interpreted as being greater than *I*_situ_ due to the combined effect of the coefficients *C*_ν_ and *C*_shut-in_. In addition, this upper bound seems to explain the centimeter-scale laboratory experiments presented in this study (cyan triangles; see Supplementary Text) and the meter-scale laboratory experiments of Cebry *et al.* (*51*) (red triangles) relatively well. The former experiments were carried out under almost identical conditions to the ones of Passelègue *et al.* (*50*), whereas the latter ones were conducted in a similar saw-cut granitic fault with hydraulic properties that are close to the ones of Passelègue *et al.*’s (*50*) fault at the lower confining pressures of this latter one.

At the large scale of industrial fluid injections, we consider one of the best-documented field cases: the 1993 hydraulic stimulation at the Soultz geothermal site in France (*3*). The hydraulic transmissivity associated with the 550-m open-hole section stimulated during the test has been estimated to experience a 200-fold increase as a consequence of the two fluid injections conducted, giving us a possible range of ~10^−14^ to 2 × 10^−12^ m^3^ (*62*). However, the smallest value of *kw* represents only the very short, initial part of the injection (*62*). Therefore, a possible variation between 5 × 10^−14^ and 2 × 10^−12^ m^3^ seems a more reasonable range to consider within the assumptions of our model, which assumes a constant transmissivity. On the other hand, the hydraulic diffusivity has substantial uncertainties due to the single-well nature of the hydraulic data in contrast to the double-well measurements used, for instance, by Passelègue *et al.* (*50*) in the laboratory. We consider a range of values for α from 0.01 to 0.1 m^2^/s, which is consistent with estimates derived from microseismicity migration (*46*) and aseismic fracture slip (*24*). Assuming a water dynamic viscosity of η = 2 × 10^−4^ Pa · s, which is representative of the temperature conditions within the reservoir (*63*), we estimate *wS* to fall within the range of 5 × 10^−8^ to 10^−7^ m/Pa. With these estimates, we calculate a representative maximum value for the in situ factor in this field test to be roughly *I*_situ_ ∼ 10^9^ N · m^−7/2^. As shown in Fig. 5, the resulting upper limit aligns very well with the field data (circles), providing an effective upper limit for the hectometer to kilometer-scale rupture cases composing the dataset. Furthermore, this simplified, order-of-magnitude analysis suggests that the behavior of the upper limit we observe from the laboratory to the reservoir scale, namely, the decrease in the factor *N* with increasingly larger volume and moment release scales, might be primarily controlled by an increase in fault zone storativity. This observation is consistent with the fact that fault zone width (*w*) alone in our dataset varies considerably, ranging from fracture hydraulic apertures on the order of tens to hundreds of micrometers in the centimeter-scale experimental fault of Passelègue *et al.* (*50*) to values in field cases that are likely similar to those of some actual geological faults, possibly reaching tens to hundreds of meters (*55*, *56*). Moreover, the upper limit for intermediate scales (in situ experiments; square symbols in Fig. 5) is characterized by a value of *N* (or *I*_situ_) that is approximately in the middle of the values that provide an upper limit for the laboratory and field data, suggesting that the increase in fault zone storativity with larger scales could be a general explanation for the trend observed throughout the entire dataset.

Next, Fig. 6 presents a comparison of our scaling relation for the maximum rupture run-out distance (Eq. 5) with the estimated rupture run-out distances for the same injection-induced aseismic slip events as in Fig. 5. To facilitate the interpretation, we similarly define the factor $M=S_{\nu }\cdot S_{\text{shut}-\text{in}}\cdot A_{\text{situ}}$ accounting for all effects other than the injected fluid volume, so that Eq. 5 becomes simply $R_{\text{max}}=M\sqrt{V_{\text{tot}}}$. We plot three values of *M* in Fig. 6 that collectively form an upper bound for the observed data across the varying scales of injection volume and rupture run-out distance within the dataset. The effects of Δτ_r−0_ and *wS* are now of similar order, as *R*_max_ scales alike with the background stress change, $R_{\text{max}}\propto \Delta \tau_{r-0}^{-1/2}$, and fault zone storativity, $R_{\text{max}}\propto {\left(\mathrm{wS}\right)}^{-1/2}$. Considering the same range of values for *f*_r_, Δτ_r−0_, and *wS* discussed previously, we calculate a representative maximum value for *A*_situ_ ∼ 100 m^−1/2^ for the experiments of Passelègue *et al.* (*50*) and *A*_situ_ ∼ 10 m^−1/2^ for the Soultz case. As shown in Fig. 6, these values are of the same order of magnitude as the upper limits (factor *M*) for the laboratory and field data, respectively. Furthermore, we observe a similar trend to that in Fig. 5: a clear scale dependency of the factor *M* with increasing injection volumes and rupture run-out distances. This scale dependency is predominantly controlled by in situ conditions (*A*_situ_), particularly fault zone storativity (*wS*).

Last, we emphasize that the values obtained for *A*_situ_ are consistent with the λ_r_ ≫ 1 regime, which forms the basis of our upper-limit scaling relations. During the injection stage, the condition that *R*(*t*) ≫ *L*(*t*) at any time *t* can also be expressed as a criterion for the variable injection flow rate *Q*(*t*). The instantaneous volume-average injection flow rate, $Q_{\text{avg}}\left(t\right)=\left(1/t\right)\int_{0}^{t}Q\left(t\prime \right)dt\prime$, equal to *V*(*t*)/*t*, must satisfy (see Eq. 29 in Materials and Methods) the following relationship with in situ conditions

$$\sqrt{Q_{\text{avg}}\left(t\right)}=\sqrt{\frac{V\left(t\right)}{t}}\gg \sqrt{4\alpha }/A_{\text{situ}}$$ (11)

Moreover, at the shut-in time *t*_s_, Eq. 11 implies the condition $A_{\text{situ}}\gg \sqrt{4\alpha /Q_{\text{avg}}}$, where $Q_{\text{avg}}=V_{\text{tot}}/t_{s}$ represents the volume-average injection flow rate of a given operation. In the inset of Fig. 6, we plot the quantity $\sqrt{4\alpha /Q_{\text{avg}}}$ as a function of fault zone hydraulic diffusivity α for a range of injection flow rates typical of industrial operations (depicted as the gray region between the two black solid lines). The red dashed line corresponds to a value of *A*_situ_ equal to 10 m^−1/2^, which is representative of the upper limit for industrial fluid injections in the main plot. The inset confirms that the relationship λ_r_ ≫ 1 is satisfied for the upper limit of industrial fluid injections shown in both Figs. 6 and 5, as the value of *I*_situ_ in Fig. 5 is derived using the same set of parameters. A similar analysis using characteristic values for the laboratory data can be conducted with analogous results.

## DISCUSSION

Our results provide a rupture mechanics–based estimate for the maximum rupture run-out distance, moment release, and magnitude of injection-induced slow slip events. Moreover, the dependence of our scaling relations on in situ conditions and injected fluid volume allows us to explain variations in rupture sizes and moment releases resulting from fluid injections that span more than 12 orders of magnitude of injected fluid volume. While our scaling relation for the maximum aseismic rupture run-out distance is the first of its kind, McGarr and Barbour (*64*) suggested in a prior work that for the moment release, the relation for the cumulative moment $\Sigma M_{0}=2\mu V_{\text{tot}}$ that was originally proposed by McGarr (*28*) for regular earthquakes also accounts for aseismic slips. It is, thus, pertinent to discuss their scaling relation in light of our findings.

We first note that in testing their relation, McGarr and Barbour (*64*) incorporated numerous data points of aseismic moment release and injected volume into a dataset characterized by otherwise only regular earthquakes. All of these aseismic slip events come from laboratory experiments of hydraulic fracturing (*65*), except for one single data point that stems from direct measurements of injection-induced aseismic slip during an in situ experiment (*6*). The mechanics of hydraulic fractures (*66*), however, differs substantially from its shear rupture counterpart. The moment release by hydraulic fractures scales linearly with the injected fluid volume, simply because the integral of the fracture width over the crack area is equal to the fracture volume. The latter is approximately equal to the injected volume under common field conditions, namely, negligible fluid leak-off and fluid lag (*66*). In our study, we discarded these hydraulic-fracturing data points because they correspond to a different phenomenon. The remaining data point of McGarr and Barbour (*64*), which corresponds to a fluid-driven shear rupture (*6*), is retained in our dataset albeit with a certain degree of uncertainty based on moment release estimates provided by more recent studies (see Supplementary Text).

In terms of modeling assumptions, one of the most important differences between McGarr’s and ours is that we account for the potential for aseismic ruptures to propagate beyond the fluid-pressurized region (λ_r_ ≫ 1). This regime, which forms the basis for our scaling relations for *R*_max_ and $M_{0}^{\text{max}}$, is not allowed by construction in McGarr’s model due to his assumption that any fault slip induced by the fluid injection must be confined within the region where pore fluids have been effectively pressurized because of the injection (*28*). We emphasize that aseismic ruptures breaking nonpressurized fault regions are a possibility that always emerges when incorporating rupture physics in a model (*44*), even in the absence of frictional weakening owing simply to long-range elastostatic stress transfer effects (*20*). Moreover, such a regime has already been directly observed in laboratory experiments (*51*) and inferred to have occurred during in situ experiments (*6*, *20*) and industrial fluid injections for reservoir stimulation (*21*). Furthermore, as a natural consequence of incorporating rupture physics in our model, we obtain a dependence of the moment release on the background stress state and fault frictional parameters. McGarr’s model is, in contrast, insensitive to these physical quantities, which largely control the release of elastic strain energy during rupture propagation. Another important distinction between the two models is that McGarr’s relies uniquely on the capacity of the rock bulk to elastically deform and volumetrically shrink to accommodate the influx of fluid mass from the injection, unlike our model, which accounts for bulk, fluid, and pore compressibilities within the fault zone via the so-called oedometric storage coefficient *S* (*43*).

Despite the substantial differences between the two models, it is pertinent to compare McGarr’s relation for the moment release with our compiled dataset. By doing so, we observe that McGarr’s upper bound can explain the majority of the data points, albeit with one very important exception (Fig. 5): the 2017 *M*_w_ 5.0 slow slip event in western Canada (*10*); the largest event detected thus far. Specifically, McGarr’s formula fails by predicting a maximum magnitude of 4.4 [considering *V*_tot_ = 88,473 m^3^ and assuming a shear modulus of 30 GPa (*10*)]. This magnitude is equivalent to predicting an upper limit for the moment release that is 16 times smaller than the actual moment that was inferred geodetically (*10*). This underestimation is somewhat similar to that performed by McGarr’s formula in the case of regular earthquakes: for instance, when considering the 2017 *M*_w_ 5.5 Pohang earthquake in South Korea (*15*). Our scaling relation can, conversely, explain the *M*_w_ 5.0 slow slip event in Canada and, more generally, our entire compilation of events by accounting for variations in in situ conditions such as the background stress change (Δτ_r−0_) and fault zone storativity (*wS*). Note that from a “data-fitting” perspective, the dependence of our model on *wS* and Δτ_r−0_ introduces additional degrees of freedom compared to McGarr’s formula, which depends only on the injected fluid volume and the shear modulus, a parameter that has very little variation in practice.

Our estimates of the maximum rupture run-out distance and magnitude for slow slip events may be regarded, to some extent, as an aseismic counterpart of the also rupture mechanics–based scaling relation proposed by Galis *et al.* (*32*) for regular earthquakes. Although we are describing a fundamentally different process here, the two scaling relations share the same 3/2–power law dependence on the injected fluid volume. This equal exponent arises from the similarities between the competing forces driving both slow slip events and dynamic ruptures in each model, namely, a point force load due to fluid injection and a uniform stress change behind the cohesive zone and within the ruptured fault area. In the model of Galis *et al.* (*32*), a point force–like load is imposed to nucleate an earthquake. In our model, it is the natural asymptotic form that the equivalent force associated with the fluid injection takes in the regime that produces the largest ruptures for a given injection (λ_r_ ≫ 1), which forms the basis for our scaling relations for *R*_max_ and $M_{0}^{\text{max}}$. Note that the two models differ in their storativity-like quantity. As we discussed before, we account for the capacity of the fluid, pore space, and bulk material in the fault zone to store pressurized fluids. In contrast, the model of Galis *et al.* (*32*) accounts only for the capacity of the bulk material: a property they inherited from McGarr’s model (*28*). A revision of seismic scaling relations may be required to include the notion of a more general storativity term, particularly considering the great variability in pore compressibility observed in practice (*57*–*60*), which can sometimes dominate over bulk and fluid compressibilities. Another important difference with the model of Galis *et al.* (*32*) is the uniform variation of background stress, which, in their model, is the so-called stress drop $\Delta \tau_{0-r}=\tau_{0}-f_{r}\sigma \prime_{0}$, whereas, in our case, it corresponds to the same quantity but of opposite sign, $\Delta \tau_{r-0}=f_{r}\sigma \prime_{0}-\tau_{0}$ (see Fig. 1D). Conceptually, this is a very important difference. In our model based on unconditionally stable ruptures, the stress drop is negative. We emphasize that the conditions leading to a negative stress drop and consequently to unconditionally stable slip are not solely restricted to faults with relatively low prestress (τ_0_) and/or those misaligned with the local in situ stress field. Frictional rheology can independently play an important role. For instance, empirically derived rate-and-state friction laws [(*67*) and references therein] suggest that even faults with high prestress and/or favorable orientations relative to the in situ stress field may exhibit negative stress drops due to rate-strengthening behavior.

The previous point brings us to an important issue: We considered only one of the two possible modes of aseismic slip, namely, fault ruptures that are unconditionally stable. However, injection-induced aseismic slip can also be the result of conditionally stable slip, that is, the nucleation phase preceding an otherwise dynamic rupture. The principal factor determining whether aseismic slip will develop in one way or the other is the so-called ultimate stability condition (*25*, *44*), which relates simply to the sign of the stress drop. For conditionally stable slip to occur, the initial shear stress must therefore be greater than the background residual fault strength ($\Delta \tau_{0-r}=\tau_{0}-f_{r}\sigma \prime_{0}>0$), resulting in a positive stress drop. In general, we cannot rule out that the points in the dataset of Figs. 5 and 6 correspond to either conditionally stable or unconditionally stable slip, as estimating the background stress state and fault frictional properties that are representative of the reactivated fault in the data remains extremely challenging. There is, nevertheless, at least one case in the dataset in which aseismic slip is, as a matter of fact, conditionally stable. These are the two aseismic slip events from the meter-scale laboratory experiments of Cebry *et al.* (*51*), which preceded seismic ruptures that broke the entire fault interface sample (see Supplementary Text). The scaling relations resulting from this mode of aseismic slip are therefore important and should be addressed in future studies.

Our model aimed to capture the most essential physical ingredients of unconditionally stable ruptures to provide the desired theoretical insights into the physical mechanisms controlling the maximum rupture run-out distance and magnitude of injection-induced slow slip events. To achieve this, we, however, adopted several simplifying assumptions that warrant further investigation. In particular, our model does not account for fluid leak-off from the permeable fault zone to the host rock nor for permeability enhancements associated with fault slip and/or the reduction of effective normal stress due to fluid injection. Despite these simplifications, we expect our scaling relations for *R*_max_ and $M_{0}^{\text{max}}$, which are based on the so-called nearly unstable regime (λ_r_ ≫ 1), to still provide an effective upper limit with regard to these additional factors. We think that incorporating a permeable host rock would notably decrease the injection overpressure in the fault zone compared to the impermeable case, thus decelerating rupture growth. The effect of slip-induced dilatancy, which is relatively well established (*68*, *69*), would introduce a toughening effect that would similarly slow down slip propagation from a fracture-mechanics perspective. Furthermore, as shown recently by Dunham (*69*), permeability enhancements due to both dilatancy and reduced effective normal stress are expected to be inconsequential in the regime λ_r_ ≫ 1, which is the relevant one for establishing our scaling relations for the maximum rupture run-out distance and moment release. This is due to the fact that, in this regime, most of the slipping region remains nonpressurized except for a small area near the fluid source. The strength of this small (point force–like) region remains unchanged in our model, provided that the enhanced hydraulic properties are considered as the constant ones (*69*). It is, however, important to highlight that in the so-called marginally pressurized regime (λ_r_ ≪ 1), recent findings by Dunham (*69*) suggest that a model assuming constant permeability and zero fracture energy does not necessarily provide an upper bound for the rupture size during the injection stage. This is particularly true in scenarios characterized by a strong permeability contrast between the initial permeability and the enhanced permeability within the slipping region. As Dunham (*69*) explains, a strong permeability enhancement confines fluid flow almost entirely within the sliding region. This, in turn, leads to additional pressurization during injection that accelerates the aseismic rupture compared to scenarios with no permeability changes. Last, another simplification in our model is the consideration of a single fault zone. Although this might likely be the case for the majority of the events incorporated in our dataset (*6*, *10*, *21*, *50*–*54*), in some cases, a network of fractures or faults could be reactivated instead (*3*, *63*). Recent numerical modeling studies on injection-induced aseismic slip have, however, shown that approximately the same scaling relation for the moment release predicted for a single fracture in two dimensions emerges collectively for a set of reactivated fractures belonging to a two-dimensional discrete fracture network (*45*). This is notably the case when the regime λ_r_ ≫ 1 is reached in a global, fracture-network sense. However, the generality and prevalence of this finding in three dimensions remain to be confirmed.

We notably showed that in the nearly unstable regime (λ_r_ ≫ 1), the dynamics of the rupture expansion in our upper bound configuration are controlled uniquely by the history of injected fluid volume, irrespective of any other characteristic of the injection protocol. The implications of this finding may go well beyond the ones explored in this work. For example, in hydraulic stimulation operations for the development of deep geothermal energy, microseismicity clouds, which often accompany fluid injections, are commonly used to constrain the areas of the reservoir that have been effectively stimulated. If aseismic-slip stress transfer is a dominant mechanism in the triggering of microseismicity, then our model suggests that these seismicity clouds may contain important information about the preinjection stress state and fault frictional properties, which are embedded in the factor *A*_situ_ (Eq. 3). Moreover, if the effect of the fracture energy on rupture propagation can be approximately neglected in comparison to the other two competing forces driving aseismic ruptures in our model, that is, the background stress change and fluid injection force, then our results imply that the spatiotemporal patterns of seismicity migration might be deeply connected to injection protocols via the dependence of the aseismic slip front dynamics on the square root of the cumulative injected fluid volume. This could be used, for instance, to identify from injection-induced seismicity catalogs under what conditions aseismic slip stress transfer may become a potentially dominant triggering mechanism due to this unique spatiotemporal footprint, which differs notably from the ones emerging from other triggering mechanisms such as pore pressure diffusion and poroelastic stressing (*70*, *71*). Similarly, our model could potentially be applied to the study of natural seismic swarms, where fluid flow and aseismic slip processes are sometimes thought to be the driving forces behind their observed dynamics (*72*, *73*). Last, our model could also be used to understand slow slip events occurring at tectonic plate boundaries in many subduction zones worldwide. The fundamental mechanics of slow slip events remains debated (*74*), yet multiple recent observations suggest that their onset and arrest might be spatially and temporally correlated with transients in pore-fluid pressure (*75*–*77*).

Last, we emphasize that our investigation has focused on constraining the rupture size and moment release of purely aseismic injection-induced ruptures. However, in some instances, seismic or microseismic events may release a substantial portion of the elastic strain energy stored in the medium. In this study, we incorporated in our compilation of events only cases where the seismic contribution to the moment release is thought to be orders of magnitude smaller than the aseismic part. From a mechanics perspective, this aimed to exclude events where the stress transfer from frictional instabilities could considerably influence the dynamics of the slow rupture under consideration, thereby ensuring a robust comparison between the data and the scaling relations of our model. Future studies should therefore focus on understanding what physical factors govern the partitioning between aseismic and seismic slips during injection operations. Our work, in this sense, contributes to this possibility by providing an upper limit to the previously unexplored aseismic end-member. Together with prior works on purely seismic ruptures, we believe that this offers a starting point to examine slip partitioning during injection-induced fault slip sequences: a crucial step toward advancing our physical understanding of the seismogenic behavior of reactivated faults and the associated seismic hazard.

## MATERIALS AND METHODS

### Time-dependent upper bound model for unconditionally stable ruptures driven by injection at a constant flow rate

We define the axisymmetric overpressure due to the injection as $\Delta p\left(r,t\right)=p\left(r,t\right)-p_{0}$, with *p*_0_ as the uniform background pore pressure. During the injection stage (*t* ≤ *t*_s_), the overpressure is given by $\Delta p\left(r,t\right)=\Delta p_{*}\cdot E_{1}\left(r^{2}/4\alpha t\right)$ for an injection at constant flow rate *Q*_0_ (*78*), where $\Delta p_{*}=Q_{0}\eta /4\pi \text{kw}$ is the intensity of the injection with units of pressure, α is the fault hydraulic diffusivity, η is the fluid dynamic viscosity, the product *kw* is the so-called fault hydraulic transmissivity, and *E*_1_ is the exponential integral function. The fracture-mechanics energy balance for a quasi-static circular rupture propagating on a slip-weakening fault was presented in (*25*) (equation 14 therein). In our upper bound configuration here, neglecting the fracture energy spent during rupture propagation implies that the stress intensity factor due to injection overpressure, *K*_p_, must equal the stress intensity factor due to the uniform background stress change, *K*_τ_. This can be written as the following expression describing the evolution of the rupture radius *R* with time

$$K_{p}=K_{\tau }\Leftrightarrow \frac{2}{\sqrt{\pi }}\frac{f_{r}\Delta p_{*}}{\sqrt{R\left(t\right)}}\int_{0}^{R\left(t\right)}\frac{E_{1}\left(r^{2}/4\alpha t\right)}{\sqrt{R{\left(t\right)}^{2}-r^{2}}}rdr=\frac{2}{\sqrt{\pi }}\Delta \tau_{r-0}\sqrt{R\left(t\right)}$$ (12)

where $\Delta \tau_{r-0}=f_{r}\sigma \prime_{0}-\tau_{0}$ is the background stress change. In Eq. 12, the integral term of the left-hand side is the stress intensity factor associated with an influx of potential energy toward the rupture front, which becomes available for the rupture to grow owing to the sole effect of overpressure due to the injection. Conversely, the term of the right-hand side is the stress intensity factor due to the background stress change, which is responsible alone for resisting rupture advancement. Nondimensionalization of Eq. 12 shows that the competition between the two energy terms is quantified by a single dimensionless number, the so-called residual stress–injection parameter $T_{r}=\Delta \tau_{r-0}/f_{r}\Delta p_{*}$, introduced first in (*25*). Moreover, Eq. 12 admits analytical solution in the form: $R\left(t\right)=\lambda_{r}L\left(t\right)$ (*23*), with the asymptotes $\lambda_{r}≃1/\sqrt{2T_{r}}$ for nearly unstable ruptures ($\lambda_{r}\gg 1,T_{r}\ll 1$), and $\lambda_{r}≃e^{\left(2-\gamma -T_{r}\right)/2}/2$ for marginally pressurized ruptures ($\lambda_{r}\ll 1,T_{r}∼10$). To highlight how important it is to analyze the end-member cases of nearly unstable (λ_r_ ≫ 1) and marginally pressurized (λ_r_ ≪ 1) ruptures throughout this work, we refer to their asymptotes for λ_r_ plotted in Fig. 2B, which nearly overlap and, thus, quantify together almost any rupture scenario. The analytical solution for λ_r_ [equation 21 in (*23*); black solid line in Fig. 2B] was first derived by Sáez *et al.* (*23*) for a fault interface with a constant friction coefficient. Here, in our upper bound configuration, the mathematical solution is identical to the one presented in (*23*), provided that the constant friction coefficient *f* in (*23*) is understood as the residual value *f*_r_ of the slip-weakening friction law.

To link the evolution of the rupture radius *R*(*t*) and the injected fluid volume *V*(*t*) in the nearly unstable regime (λ_r_ ≫ 1), we use the asymptote $R\left(t\right)≃\left(1/\sqrt{2T_{r}}\right)L\left(t\right)$ in combination with the following expressions for the residual stress–injection parameter $T_{r}=\Delta \tau_{r-0}/f_{r}\Delta p_{*}$, overpressure intensity $\Delta p_{*}=Q_{0}\eta /4\pi \text{kw}$, overpressure front $L\left(t\right)=\sqrt{4\alpha t}$, hydraulic diffusivity α = *k*/Sη, and injected fluid volume *V*(*t*) = *Q*_0_*t*. By doing so, we arrive at Eq. 3. For noncircular ruptures (ν ≠ 0), building upon the work of Sáez *et al.* (*23*) for a constant friction coefficient, we obtain that the rupture front of our upper bound model is well approximated by an elliptical shape that becomes more elongated for increasing values of ν and decreasing values of $T_{r}$, with a maximum aspect ratio of 1/(1 − ν) when $T_{r}\ll 1$ and a minimum aspect ratio of (3 − ν)/(3 − 2ν) when $T_{r}∼10$. Other features of Sáez *et al.*’s model (*23*) such as the invariance of the rupture area with regard to Poisson’s ratio and the numerically derived asymptotes for the quasi-elliptical fronts are also inherited here in the upper bound model. In the shut-in stage (*t* > *t*_s_), the overpressure is obtained by superposition simply as $\Delta p\left(r,t\right)=\Delta p_{*}\cdot \left[E_{1}\left(r^{2}/4\alpha t\right)-E_{1}\left(r^{2}/4\alpha \left(t-t_{s}\right)\right)\right]$. The spatiotemporal evolution of overpressure has been studied in detail in (*24*). Moreover, as discussed in the “Physical model and upper bound rationale for unconditionally stable ruptures” section, we reduce the upper bound model in the shut-in stage to a fault responding with a constant friction coefficient equal to *f*_r_. Hence, our upper bound model inherits all the results obtained by Sáez and Lecampion (*24*) who investigated extensively the propagation and arrest of postinjection aseismic slip on a fault obeying a constant friction coefficient. In particular, we take advantage of their understanding of the propagation and arrest of the slip front that ultimately determines the maximum size of unconditionally stable ruptures in our upper bound model. In the present work, we have expanded the work of Sáez and Lecampion (*24*) to account for an examination of the previously unknown evolution of the moment release during the shut-in stage (Fig. 4).

### Asymptotics of moment release for nearly unstable and marginally pressurized circular ruptures driven by injection at a constant flow rate

The scalar moment release *M*_0_ at a given time *t* is given by (*79*)

$$M_{0}\left(t\right)=\mu \iint_{A_{r}\left(t\right)}\delta \left(x,y,t\right)dxdy$$ (13)

where μ is the bulk shear modulus, δ is the current slip distribution, and *A*_*r*_ is the current rupture area. To calculate the time-dependent slip distribution in the circular rupture case, we consider the quasi-static relation between fault slip δ and the associated elastic change of shear stress Δτ within an axisymmetric circular shear crack (*80*)

$$\delta \left(r,t\right)=\frac{4R\left(t\right)}{\pi \mu }\int_{\bar{r}}^{1}\frac{\xi d\xi }{\sqrt{\xi^{2}-{\bar{r}}^{2}}}\int_{0}^{1}\frac{\Delta \tau \left[\mathrm{s\xi R}\left(t\right),t\right]sds}{\sqrt{1-s^{2}}}$$ (14)

where $\bar{r}=r/R\left(t\right)$ is the normalized radial coordinate. Equation 14 was originally derived for an internally pressurized tensile circular crack with axisymmetric load (*80*). Nevertheless, under the assumptions of unidirectional slip with axisymmetric magnitude and a Poisson’s ratio ν = 0, the shear crack problem is mathematically equivalent on the fault plane to its tensile counterpart (*20*): crack opening being δ and crack-normal stress change being Δτ. In the limiting regime of a rupture propagating with zero fracture energy and at the residual friction level *f*_r_, the change of shear stress is simply

$$\Delta \tau \left(r,t\right)=\tau_{0}-f_{r}\left[\sigma \prime_{0}-\Delta p\left(r,t\right)\right]=f_{r}\Delta p\left(r,t\right)-\Delta \tau_{r-0}$$ (15)

where $\Delta \tau_{r-0}=f_{r}\sigma \prime_{0}-\tau_{0}$ is the background stress change. Hence, for injection at a constant volumetric rate *Q*_0_, the spatiotemporal evolution of slip for the end-member cases of nearly unstable (λ_r_ ≫ 1) and marginally pressurized (λ_r_ ≪ 1) ruptures turn out to be identical to the ones determined by Sáez *et al.* (*23*) for their so-called critically stressed regime [equation 26 in (*23*)] and marginally pressurized regime [equation 25 in (*23*)], respectively, as long as we interpret their constant friction coefficient *f* as *f*_r_. Here, we write the resulting self-similar slip profiles in a more convenient dimensionless form

$$\delta \left(r,t\right)/\delta_{*}\left(t\right)=D\left[r/R\left(t\right)\right],\;\text{with}\;\delta_{*}\left(t\right)=\left\{ \begin{array}{c} \Delta \tau_{r-0}R\left(t\right)/\mu \;\text{when}\;\lambda_{r}\gg 1\\ f_{r}\Delta p_{*}R\left(t\right)/\mu \;\text{when}\;\lambda_{r}\ll 1 \end{array}\right.$$ (16)

and

$$D\left(x\right)=\left\{\begin{array}{c} \left(4/\pi \right)\left[\text{arccos}\left(x\right)/x-\sqrt{1-x^{2}}\right]\;\text{when}\;\lambda_{r}\gg 1\\ \left(8/\pi \right)\left[\sqrt{1-x^{2}}-x\cdot \text{arccos}\left(x\right)\right]\;\text{when}\;\lambda_{r}\ll 1 \end{array}\right.$$ (17)

Note that in the nearly unstable regime (λ_r_ ≫ 1), we recast equation 26 in (*23*) using the expressions *L*(*t*) = *R*(*t*)/λ_r_ and $\lambda_{r}≃1/\sqrt{2T_{r}}$. The nearly unstable asymptote for fault slip is plotted in Fig. 3B and compared to the numerical solution.

Integration of the self-similar slip profiles via Eq. 13 leads to the asymptotes for the moment release during the injection stage given in the “Maximum moment release and magnitude” section: $M_{0}\left(t\right)=\left(16/3\right)\Delta \tau_{r-0}R{\left(t\right)}^{3}$ when λ_r_ ≫ 1, and $M_{0}\left(t\right)=\left(16/9\right)f_{r}\Delta p_{*}R{\left(t\right)}^{3}$ when λ_r_ ≪ 1. It is worth mentioning that the slip distribution of nearly unstable ruptures has a singularity (of order 1/*r*) at *r* = 0. Strictly speaking, this asymptote corresponds to the solution of the so-called outer problem, which is defined at distances *r* ≫ *L*(*t*). An interior boundary layer must be resolved at distances *r* ∼ *L*(*t*) to obtain the finite slip at the injection point, which scales as $\delta_{c}\left(t\right)=f_{r}\Delta p_{*}L\left(t\right)/\mu$ (see Fig. 3B) (*23*). Nevertheless, this boundary layer has no consequences in estimating the moment release. The integrand in Eq. 13 for such a slip distribution is nonsingular so that after taking the limit *L*(*t*)/*R*(*t*) → 0, one effectively recovers the actual asymptote for the moment release. The details of the interior boundary layer are therefore irrelevant to the calculation of *M*_0_ in this limit.

### Relation between fluid injection force and injected fluid volume for arbitrary fluid sources

Under the assumptions of our model, the displacement field **u** induced by the fluid injection into the poroelastic fault zone is irrotational ∇ × **u** = 0 (*41*). Therefore, the variation in fluid content ζ, which corresponds to the change of fluid volume per unit volume of porous material with respect to an initial state (here, *t* = 0), satisfies the following constitutive relation with the pore-fluid overpressure Δ*p* [equation 96 in (*42*)]

ζ=SΔp (18)

where *S* is the so-called oedometric storage coefficient representing the variation of fluid content caused by a unit pore pressure change under uniaxial strain and constant normal stress in the direction of the strain (*43*), here, the *z* axis (Fig. 1C). *S* accounts for the effects of fluid, pore, and bulk compressibilities of the fault zone and is equal to (*42*)

$$S=\frac{1}{M}+\frac{b^{2}\left(1-2\nu \right)}{2\left(1-\nu \right)\mu }$$ (19)

where *M* is the Biot’s modulus and *b* is the Biot’s coefficient.

To obtain the cumulative injected fluid volume at a given time, *V*(*t*), we just sum up changes in fluid volume all over the spatial domain of interest, say Ω, at a given time *t*, that is, $V\left(t\right)=\int_{\Omega }\zeta d\Omega$. In our model, the fluid flow problem is axisymmetric, and the fault zone width *w* is uniform, such that the differential of the volume is simply dΩ = 2π*wr*d*r* in cylindrical coordinates. With these definitions, we can now integrate (Eq. 18) over the entire fault zone volume to obtain the following expression for the injected fluid volume valid for an arbitrary fluid injection

$$V\left(t\right)=\mathrm{wS}\cdot 2\pi \int_{0}^{\infty }\Delta p\left(r,t\right)rdr$$ (20)

By defining the normal force induced by the fluid injection over the slip surface (simply equal to the integral of the overpressure over the fault plane) as

$$F\left(t\right)=2\pi \int_{0}^{\infty }\Delta p\left(r,t\right)rdr$$ (21)

we arrive at the following relation between the fluid injection force and injected fluid volume

$$F\left(t\right)=\frac{V\left(t\right)}{\mathrm{wS}}$$ (22)

Expressions of a similar kind to Eq. 22 have been reported in previous studies (*28*, *81*, *82*). For example, McGarr (*28*) considered a similar relation except that his storativity-like term is the inverse of the elastic bulk modulus. Garagash (*82*) also proposed a similar expression to Eq. 22 but accounting only for pore compressibility. Last, the relation considered by Shapiro *et al.* (*81*) is the closest to our expression, including the oedometric storage coefficient.

### Evolution of rupture radius and moment release for nearly unstable circular ruptures driven by arbitrary fluid injections

As discussed in the “Dynamics of unconditionally stable ruptures and maximum rupture run-out distance” section, nearly unstable ruptures (λ_r_ ≫ 1) produce the largest events for a given injection, making them the natural basis for calculating the maximum rupture size *R*_max_ and moment release $M_{0}^{\text{max}}$. Here, we generalize the relations for the rupture radius *R*(*t*) and moment release *M*_0_(*t*) of nearly unstable circular ruptures with the injected fluid volume *V*(*t*), Eqs. 3 and 7, respectively, to account for arbitrary fluid injections during the injection stage. Let us first note that the reduction of fault strength due to fluid injection in the so-called outer problem [*r* ≫ *L*(*t*)] can be effectively approximated as a point force [e.g., (*23*, *44*)]

$$f_{r}\Delta p\left(r,t\right)\approx f_{r}F\left(t\right)\frac{\delta^{\text{dirac}}\left(r\right)}{2\pi r}=f_{r}\frac{V\left(t\right)}{\mathrm{wS}}\frac{\delta^{\text{dirac}}\left(r\right)}{2\pi r}$$ (23)

where *F*(*t*) is the fluid injection normal force (Eq. 21), which is related to the cumulative injected fluid volume via Eq. 22. Substituting Eq. 23 into the stress change (Eq. 15) and then the latter into the double integral representing the quasi-static elastic equilibrium (Eq. 14), we obtain, upon evaluating those integrals, an asymptotic upper bound for the spatiotemporal evolution of fault slip as

$$\delta \left(r,t\right)=\frac{4}{\pi }\frac{\Delta \tau_{r-0}}{\mu }R\left(t\right)\left[\frac{f_{r}V\left(t\right)}{2\pi \mathrm{wS}\Delta \tau_{r-0}}\frac{\text{arccos}\left(r/R\left(t\right)\right)}{r\cdot R\left(t\right)}-\sqrt{1-{\left(r/R\left(t\right)\right)}^{2}}\right]$$ (24)

Note that Eqs. 22 and 23 are valid for fluid injections that are entirely arbitrary. However, by substituting the stress change (Eq. 15) into the elastic equilibrium (Eq. 14), we assumed that the shear stress in the sliding region, 0 ≤ *r* ≤ *R*(*t*), equals the fault shear strength at any time *t*. That is, we assumed crack-like propagation. This is the reason why the degree of arbitrariness of the fluid injection is associated with maintaining crack-like behavior during the injection stage. In the shut-in stage, we account for the dynamics of pulse-like ruptures that emerge upon stopping the injection for the particular case of injection at a constant flow rate. However, during injection, the variable injection flow rate must be such that a transition from crack-like to pulse-like rupture is prevented. The necessary and sufficient conditions for the fluid source to produce a pulse are currently unknown.

The propagation condition for a rupture with negligible fracture energy is given by Eq. 12. This condition can be alternatively written in terms of the slip behavior near the rupture front as (*83*)

$$\underset{r\to R{\left(t\right)}^{-}}{\text{lim}}\frac{\partial \delta (r,t)}{\partial r}\sqrt{R\left(t\right)-r}=0$$ (25)

The previous equation imposes a constraint on the slip distribution (Eq. 24) that can be also seen, in a limiting sense, as eliminating any stress singularity at the rupture front. By differentiating Eq. 24 with respect to *r* and then applying the propagation condition (Eq. 25), we obtain the following relation, which is valid for arbitrary fluid injections as long as crack-like propagation holds

$$R\left(t\right)=\sqrt{\frac{f_{r}V\left(t\right)}{2\pi \mathrm{wS}\Delta \tau_{r-0}}}$$ (26)

Equation 26 is identical to Eq. 3, $R\left(t\right)=A_{\text{situ}}\sqrt{V\left(t\right)}$ in the “Dynamics of unconditionally stable ruptures and maximum rupture run-out distance” section. The latter was originally derived for injection at a constant flow rate. It, thus, represents a generalization of the relation between the evolution of the rupture radius and the cumulative injected fluid volume (Eq. 3) for arbitrary fluid injections.

Furthermore, Eq. 26 can be recast to use the so-called stress-injection parameter, extensively used in prior works for injection at constant flow rate (*23*–*25*). Let us define a time-dependent amplification factor for a frictional rupture with zero fracture energy and variable injection flow rate, $\lambda_{r}\left(t\right)=R\left(t\right)/L\left(t\right)$ and the instantaneous volume-average flow rate of an injection

$$Q_{\text{avg}}\left(t\right)=\frac{1}{t}\int_{0}^{t}Q\left(t\prime \right)dt\prime =\frac{V\left(t\right)}{t}$$ (27)

Combining Eqs. 26 and 27, together with $L\left(t\right)=\sqrt{4\alpha t}$ and the oedometric storage coefficient written as *S* = *k*/ηα, we obtain the following expression for the time-dependent amplification factor

$$\lambda_{r}\left(t\right)=\frac{1}{\sqrt{2T_{r}\left(t\right)}},\;\text{with}\;T_{r}\left(t\right)=\frac{\Delta \tau_{r-0}}{f_{r}\Delta p_{*}\left(t\right)}\;\text{and}\;\Delta p_{*}\left(t\right)=\frac{Q_{\text{avg}}\left(t\right)\eta }{4\pi \mathrm{kw}}$$ (28)

Equation 28, thus, generalizes the asymptotic solution for nearly unstable ruptures driven by constant injection flow rates, $\lambda_{r}=1/\sqrt{2T_{r}}$ (Fig. 2B and see Eq. 1 for the definition of $T_{r}$ for constant flow rate), to account for arbitrary fluid injection scenarios. While retaining the same structure, this generalized solution incorporates the instantaneous volume-average injection flow rate (Eq. 27) as the relevant flow rate of the problem, resulting in a time-dependent overpressure intensity Δp_*_(*t*) and a time-dependent residual stress–injection parameter $T_{r}\left(t\right)$. For the specific case of constant injection flow rate *Q*(*t*) = *Q*_0_, Eq. 28 effectively reduces to the solution found in (*23*), now recognized as a particular example of a broader class of asymptotic solutions for λ_r_ ≫ 1 and zero fracture energy.

Now, it is entirely possible that, for a variable injection flow rate *Q*(*t*), the condition characterizing nearly unstable ruptures, *R*(*t*) ≫ *L*(*t*), may not be satisfied at a given time *t*. To apply Eqs. 26 and 28, it is essential to verify that the time-dependent residual stress–injection parameter, $T_{r}\left(t\right)$, remains sufficiently small [$T_{r}\left(t\right)\ll 1$]. In practice, however, the nearly unstable asymptotic solution provides a somewhat reliable approximation of the slip front position, even when λ_r_(*t*) approaches unity. For instance, the analytical solution for constant flow rate injection shown in Fig. 2B illustrates that, at λ_r_ = 1, the asymptote for λ_r_ ≫ 1 approximates the exact solution with a relative error of only 8%. This suggests that Eqs. 26 and 28 may be useful even when $T_{r}\left(t\right)∼0.5$.

Moreover, the condition $T_{r}\left(t\right)\ll 1$ can be reformulated into a more practical constraint on fluid injection, which can be expressed in terms of in situ conditions as follows

$$\sqrt{Q_{\text{avg}}\left(t\right)}=\sqrt{\frac{V\left(t\right)}{t}}\gg \frac{\sqrt{4\alpha }}{A_{\text{situ}}}=\sqrt{\frac{8\pi \alpha \mathrm{wS}\Delta \tau_{r-0}}{f_{r}}}$$ (29)

with *A*_situ_ defined in Eq. 3. This is the reasoning behind Eq. 11.

Note that Eq. 26 (and, thus, Eq. 28) can be alternatively derived through the rupture propagation condition imposed over the stress change (Eq. 12). It only takes to replace the particular overpressure solution for injection at a constant volumetric rate, $\Delta p\left(r,t\right)=\Delta p_{*}E_{1}\left(r^{2}/4\alpha t\right)$, by the more general point force representation in the nearly unstable regime (Eq. 23). Here, we report the derivation based on the slip distribution because it makes now the calculation of the moment release for arbitrary fluid injections straightforward. By substituting Eq. 26 into Eq. 24, we obtain the slip distribution satisfying the zero fracture energy condition. Upon integrating the resulting slip profile via Eq. 13, we obtain the following expression for the moment release

$$M_{0}\left(t\right)=\frac{16}{3{\left(2\pi \right)}^{3/2}}\frac{V{\left(t\right)}^{3/2}}{\sqrt{\Delta \tau_{r-0}}}{\left(\frac{f_{r}}{\mathrm{wS}}\right)}^{3/2}$$ (30)

which is identical to Eq. 7, thus demonstrating that the relation between the moment release, in situ conditions (*I*_situ_), and injected fluid volume (Eq. 7) holds for arbitrary fluid injections.

Last, we note a relevant assumption underlying the upper bound rationale of our model, which relies on the following property of unconditionally stable ruptures: The effect of the fracture energy in the front-localized energy balance must diminish as the rupture grows and, ultimately, become negligible (*25*). Although this is certainly valid even in the case of arbitrary fluid injections, it relies on an implicit assumption of the slip-weakening model, namely, the fracture energy being constant. Our theoretical framework allowed, in principle, to account for nonconstant and nonuniform fracture energy. We do not account for fracture energy heterogeneity for the same reason that we do not account for stress or other kinds of heterogeneities in our model: We aim to provide fundamental, first-order insights into the problem at hand. Moreover, we also do not consider a possible scale dependence of the fracture energy. The scale dependency of fracture energy for seismic ruptures is a topic of active research [(*84*) and references therein]. Although we do expect this phenomenon to be also present in aseismic ruptures, to the best of our knowledge, there is currently no experimental or observational evidence suggesting this behavior for slow frictional ruptures. Therefore, we choose not to explore the theoretical implications of this potential physical factor at this stage.

### Numerical methods

All the numerical calculations in this study have been conducted via the boundary element–based method described in (*23*). For the general case of noncircular ruptures, we use the fully three-dimensional method presented in (*23*). For the particular case of axisymmetric, circular ruptures, we use a more efficient axisymmetric version of the method presented in (*24*).

## Supplementary Materials

### The PDF file includes:

Supplementary Text Fig. S1 Legend for dataset S1 References
