# Supplementary material for: Maximum size and magnitude of injection-induced slow slip events
Source: Sci Adv. 2025 May 7;11(19):eadq0662. doi: 10.1126/sciadv.adq0662 (PMC12057678; doi:10.1126/sciadv.adq0662)
Supplement: Supplementary file 1 — Supplementary Text Fig. S1 Legend for dataset S1 References [file sciadv.adq0662_sm.pdf]

Science Advances
AAAS

Supplementary Materials for

Maximum size and magnitude of injection-induced slow slip events

Alexis Sáez *et al.*

Corresponding author: Alexis Sáez, saez@caltech.edu

Sci. Adv. **11**, eadq0662 (2025)
DOI: 10.1126/sciadv.adq0662

The PDF file includes:

Supplementary Text
Fig. S1
Legend for dataset S1
References

Other Supplementary Material for this manuscript includes the following:

Dataset S1

Supplementary Text

In the following sections, we provide the references and calculations behind the dataset of injection-induced aseismic slip events used in this study. For some events, the moment release, rupture run-out distance (defined as the maximum distance from the aseismic slip front to the injection point), and injected fluid volume are reported directly by the original authors. In other cases, these quantities were estimated based on the assumptions outlined below. Additionally, we include data points from previously unpublished laboratory experiments, making the dataset both compiled and produced. A summary of this dataset is provided in Data S1 (electronic .xls file). For some events in the dataset, the moment release and rupture run-out distance are estimated within a range, depicted in Figs. 5 and 6 as vertical lines connecting their maximum and minimum values. Similarly, in certain cases, the injected fluid volume is estimated within a range, represented as horizontal lines in the same figures. In Data S1, these ranges are indicated by the subscripts *max* and *min*, respectively. Additionally, in the last section, we provide a brief discussion about the injected fluid volumes considered in cases of hydraulic fracturing.

Small-scale laboratory experiments

Passelègue et al. (*50*) conducted fluid injection experiments on a saw-cut sample of andesite exhibiting negligible bulk permeability. Their experimental fault is elliptical, with a maximum length L_f = 0.08 m and a maximum width of b_f = 0.04 m (Fig. S1). Fluid injections were carried out at 90 percent of the fault's peak strength, with initial effective confining pressure ($P_{ef} = P_c - P_f$) ranging from 20 to 85 MPa (P_c = 30, 60 and 95 MPa, and P_f = 10 MPa). As highlighted by the strain gages array along the fault (Figs. S1A and S1C), an aseismic rupture nucleates near the injection borehole and propagates predominantly toward the other side of the sample where the measurement borehole is (see (*50*) for further details). At the moment in which the rupture reaches this latter edge of the experimental fault, the two sample pieces undergo a rigid-body motion. Due to the assumptions of our model which considers a rupture propagating on a fault embedded in an unbounded domain, we calculate the moment release aseismically only up to the time in which the slip front reaches that edge of the experimental fault. This time is denominated t_n (Figs. S1C and S1D). The initial time of each slow slip event, denominated as t_0, corresponds to the time

at which the fault comes back to its elastic behavior after each event. Since the experiments are conducted at a constant injection flow rate Q_0, we estimate the total injected fluid volume for each event as $V_{\text{tot}} = Q_0(t_n - t_0)$. The experiments conducted at Q_0 = 50 μL/min are the ones reported by Passelègue *et al.* (*50*). The experiments conducted at Q_0 = 10 μL/min are published for the first time in this study (see Data S1).

We estimated the macroscopic aseismic slip induced along the fault between t_0 to t_n using the macroscopic measurement of axial shortening, as given by the formula:

$$\delta = \left(\frac{D_{ax}}{L_s} - \frac{\Delta\sigma_D}{E_{ap}}\right)\frac{L_s}{\cos(\theta)}, \tag{S1}$$

where D_{ax} represents the direct measurement of axial shortening monitored using gap sensors (see (*50*) for further details), L_s is the sample length, $\Delta\sigma_D$ is the change in differential stress measured during each event, E_{ap} is the Young's modulus of the experimental apparatus, and θ is the angle between the sample axial direction and the fault plane (equal to 30 degrees).

The aseismic moment release is then computed assuming that at the end of each aseismic slip event, the entire fault interface is sliding. The moment release is thus calculated as $M_0 = \mu\delta S$, where μ = 30 GPa is the sample shear modulus, and S is the macroscopic fault surface equal to $\pi(L_f/2)(b_f/2)$. This hypothesis holds to the first order, as the estimate of δ is derived from the average strain measured along the entire sample. For the rupture run-out distance, we consider it to be equal to L_f for all events. This is consistent with the fact that each aseismic slip event is considered up to the time at which the rupture reaches the side of the sample where the measurement borehole is. The latter is the maximum possible distance from the slip front to the injection point (see Fig. S1A).

Large-scale laboratory experiments

Cebry *et al.* (*51*) reported three meter-scale experiments on a saw-cut granitic fault, denominated Cases A, B, and C in their work. The main quantity they varied throughout their experiments was the initial shear stress, from higher to lower values from Cases A to C respectively. Their experimental fault size is 3.15 m long and W = 0.3 m wide. Fluid is injected directly into the fault through a wellbore which is located right in the middle of the shortest side of the sample interface. An aseismic rupture nucleates most likely near the injection point, yet slip measurements start only

once the rupture reaches the traction-free face of the shortest side of the sample. From this time on, an *average* one-dimensional rupture is monitored while propagating along the longest side of the sample. Here, we focus on the aseismic slip phases preceding seismic events in each experiment. For Case A, a maximum aseismic slip of $\delta_A = 46.9$ µm was measured at the sensor that was closest to the injection point within a measured rupture length of approximately $L_A = 1.4$ m. Assuming a symmetric and linear distribution of slip from the maximum slip δ_A to zero at the rupture front, we estimate the moment release as $M_0 = \mu\delta_A L_A W/2$. Considering a shear modulus of 30 GPa, the estimated moment release is then 2.95×10^5 Nm. For Case B, a maximum aseismic slip of $\delta_B = 92.6$ µm was measured within a rupture extent of approximately $L_B = 1.6$ m. Likewise, we can estimate the moment release in 6.67×10^5 Nm. The experiments were conducted at a constant injection flow rate equal to 10 mL/min. Cebry *et al.* (*51*) reported that the time between the injection start and the maximum fluid pressure measured at the well during each experiment varied from 140 to 200 seconds. The instant of maximum pressure was associated with the time at which the injected fluid reached the shortest side of the sample for the first time. Starting from this moment, fluid leaks from the sample interface. In Cases A and B, the aseismic slip phases ended shortly upon reaching the instant of maximum pressure, after approximately 3 seconds and 15 seconds respectively. Since these periods are short compared to the time to reach the maximum pressure and the pressure drop associated with fluid leakage is small relative to the maximum pressure, we neglect the fluid losses in Cases A and B. Hence, we calculate a minimum and maximum injected volume that are representative of these two aseismic slip events as 10 mL/min × 143 sec = 2.4×10^{-5} m^3 and 10 mL/min × 215 sec = 3.6×10^{-5} m^3 respectively, which are reported in Data S1. For Case C, the aseismic slip phase ended around 500 seconds after reaching the maximum pressure. In principle, we think it is not possible to neglect the fluid loss here for the volume calculation. Hence, we do not consider this case in the dataset. Finally, the maximum rupture run-out distance is calculated as $L_A/2 = 0.7$ m and $L_B/2 = 0.8$ m for Cases A and B respectively. This is because the ruptures are nearly symmetric with regard to the injection point so half of the rupture length corresponds to the rupture run-out distance from the injection well.

In-situ experiments on shallow natural faults

The 2015 Guglielmi *et al.*'s experiment (*6*) in Southeastern France

In 2015, Guglielmi *et al.* (*6*) conducted an experiment of fault reactivation by fluid injection in a shallow carbonate fault system in Southeastern France, by injecting 0.95 m^3 of water at a depth of about 280 m. They monitored micro-seismicity, injection-well fluid pressure, volume rate history, and the history of induced fracture slip and opening at the injection interval. Guglielmi *et al.* concluded that most of the induced fracture slip was aseismic, with a maximum value of about 1.2 cm at the injection interval. By fitting part of the data through a simplified hydro-mechanical model including a circular rupture with complete stress drop, they estimated the associated aseismic moment release in 6.5×10^{10} Nm. For this same experiment, another estimate of the aseismic moment release may be obtained from the inverse modeling results of Bhattacharya and Viesca (*20*). They estimated the rupture radius at the moment the injection stops in approximately $R = 6.5$ m and a shear modulus of about 12 GPa. Considering a triangular distribution of slip from the measured maximum slip at the injection interval $\delta_{\text{well}} = 1.2$ cm to zero at the rupture front which we approximate as being circular, we estimate the moment release in $(\pi/3)\mu\delta_{\text{well}}R^2 \approx 6.4 \times 10^8$ Nm. This value is two orders of magnitude lower than the one estimated by Guglielmi *et al.* (*6*). The difference is essentially due to the dissimilar estimates for the rupture radius. Guglielmi *et al.* (*6*) estimated the rupture radius to be approximately 35 m, which is more than five times larger than the one estimated by Bhattacharya and Viesca (*20*). We therefore consider the estimates of moment release and rupture radius (taken as maximum rupture run-out distance) coming from these two previous studies as maximum and minimum values, in Data S1, respectively.

The 2014 De Barros *et al.*'s experiments (*53*) in Tournemire, France

In 2014, a set of in-situ experiments reported by De Barros *et al.* (*53*) took place in an underground laboratory (IRSN) in Tournemire, France. Fluids were injected into a low-permeability shale fault zone at a depth of approximately 200 m. The acquired datasets are similar to the ones obtained by Guglielmi *et al.* (*6*). Here, we consider two events for which the injected fluid volume, aseismic moment release, and rupture run-out distance are reported by De Barros *et al.* (*33*). The estimates of aseismic moment release, denominated as deformation moment in (*33*), are several orders of magnitude greater than the cumulative moment released by the monitored micro-seismicity, thus highlighting the dominance of aseismic slip in these experiments. De Barros *et al.* (*33*) provided an uncertainty range for both aseismic moment release and rupture radius (taken as maximum rupture run-out distance) of each event that we incorporate directly in our compiled

dataset, Data S1 (the original data is in figure 2 and supplementary materials in (*33*)).

The 2015 Duboeuf *et al.*'s experiments (*54*) in Rustrel, France

In 2015, a set of in-situ experiments reported by Duboeuf *et al.* (*54*) was conducted in an underground laboratory (LSBB) in Rustrel, France. Fluid injections were performed, in this case, into a fault damage zone in limestone, at a depth of about 300 m. Here, we consider six events for which the injected fluid volume, aseismic moment release, and rupture radius are also reported by De Barros *et al.* (*33*). To provide an uncertainty range for the aseismic moment release and rupture radius (taken as maximum rupture run-out distance) of each event, they considered the same kind of uncertainties as for the experiments in IRSN, Tournemire, France. We incorporate those uncertainty ranges directly in our compilation of events, Data S1 (the original data is in figure 2 and supplementary materials in (*33*)).

Large-scale fluid injections in the field

The 1993 hydraulic stimulation at the Soultz geothermal site, France

A hydraulic stimulation was conducted in two stages in 1993 at the Soultz geothermal site in France, where a total water volume of 44600 m^3 was injected into granite through a 550 m open-hole section located at depths between 2850 m and 3400 m (*3*). Cornet *et al.* (*3*) estimated fracture slips along the open-hole section of the well from ultrasonic imaging logs in up to 4.7 cm, as a result of the two hydraulic stimulation stages. Based on two scenarios of stress drop (equal to 9 and 19 MPa), they estimated the moment release associated with the inferred fracture slips in 3.6×10^{13} and 8.9×10^{12} Nm, respectively. Because the moment release by the largest seismic event recorded during the operation was estimated to be about two orders of magnitude lower than the moment release estimated from fracture slip, they concluded that the moment release during this experiment was mainly due to aseismic motions (*3*). On the other hand, by analyzing repeating earthquakes (multiplets) in one of the faults with the greatest slip, Bourouis and Bernard (*4*) provided further support to the mostly aseismic nature of the reservoir deformation during this hydraulic-stimulation experiment. From the analysis of Bourouis and Bernard, we constrain the aseismic moment release as $\sim \mu\delta_{avg}L^2$, where μ = 20 GPa (*3*), δ_{avg} is the average slip on the fault estimated from the repeaters analysis in 4 cm (*4*) which is remarkably consistent with the estimates by Cornet *et al.* (*3*) from ultrasonic imaging logs, and L is the characteristic size of the slipped fault patch. An upper

bound for L of approximately 500 m can be considered if one assumes that all the micro-seismicity (including repeaters and non-repeaters) delineating the examined fault (see figure 5 in (*4*)) has been triggered by shear loading due to aseismic slip surrounding nominally locked asperities where the micro-seismic events are hosted. On the other hand, if we consider that micro-seismicity could be also triggered ahead of the aseismic slip front due to quasi-static stress transfer and assume repeaters as evidence for aseismic slip around the repeaters source, one can estimate a lower bound for L corresponding to the minimum fault patch size that contains all the repeaters (see figure 5 in (*4*)), in approximately 350 m. This yields an aseismic moment release of 9.8×10^{13} and 2.0×10^{14} Nm for the lower and upper bounds of the slipped patch size, respectively. Taking together the estimates from the studies by Cornet *et al.* (*3*) and Bourouis and Bernard (*4*), we obtain a minimum and maximum value of M_0 as 8.9×10^{12} Nm and 2.0×10^{14} Nm, respectively, which are the two values reported in Data S1. For the rupture run-out distance, we consider a minimum value of 68 m from the rupture radius estimated by Cornet *et al.* (*3*) at their highest stress drop scenario, and a maximum value of 500 m from the scenario in which the aseismic slip patch wraps all the micro-seismicity in Bourouis and Bernard's analysis (*4*), consistently with the minimum and maximum values for the moment release.

The 2017-2018 hydraulic-fracturing-induced slow slip events in Northwestern Canada

Eyre *et al.* (*10*) have recently reported the two largest injection-induced slow slip events detected thus far. The first event of magnitude M_w 5.0 occurred in September 2017, whereas the second event of magnitude M_w 4.2 occurred approximately one year later, in October 2018. Both events were attributed to hydraulic fracturing operations into a low-permeability hydrocarbon reservoir (the so-called Montney Formation, a fine-grained siltstone) that were conducted through two horizontal wells at a depth of approximately 2 km. The total injected fluid volume associated with each of these events is 88473 m^3 and 98193 m^3, respectively (*10*). The moment release was estimated from kinematic inversions of fault slip using InSAR surface deformation measurements, resulting in shallowly-dipping (8°) thrust events that are consistent with known bedding planes within the Montney Formation (*10*). The inferred aseismic slip events were corroborated independently against large well-case deformations reported at the same depths as the events, just above the horizontal wells. The moment release was estimated in 4.24×10^{16} Nm for the largest event in 2017, and 2.09×10^{15} Nm for the smaller event in 2018. The associated maximum slips for each event are 24

cm and 11 cm, respectively. We consider their rupture run-out distances as half of the rupture size in the most elongated direction of the slipped fault patch (see figure 3a in (*10*)), namely, ≈2.5 km and ≈1.75 km respectively. This assumes that the equivalent injection point for each event would be approximately in the centroid of the rupture surface. The slow slip events were likely produced by hydraulic fractures intersecting and activating the bedding planes in shear. We do not attempt to determine an equivalent injection point from the multiple hydraulic fracturing stages. Finally, it is important to note that no seismic events were detected by the regional network (with a detection threshold of ~M1.5 (*10*)). Hence, the geodetically inferred moment releases can be reasonably assumed to correspond to virtually pure aseismic ruptures.

The 2016 hydraulic-fracturing-induced slow slip transient that likely triggered the M_w 4.1 earthquake in Alberta, Canada

In 2016, an earthquake of magnitude M_w 4.1 was triggered by hydraulic fracturing operations in an unconventional hydrocarbon reservoir in Alberta, Canada (*21*). Eyre *et al.* (*21*) investigated the triggering mechanism of this earthquake. They provided evidence for an aseismic slip event induced by hydraulic fractures that intersected (and pressurized) at the reservoir level (shales at around 3.4 km depth), frictionally stable segments of a nearby sub-vertical strike-slip fault. They suggested that this fluid-driven aseismic slip event propagated and transmitted solid stresses to distal (upward) segments of the same fault but in frictionally unstable carbonate units where the hypocenter of the M_w 4.1 earthquake was located (at about 3 km depth). Our focus here is the aseismic slip transient which developed mostly on presumably unconditionally stable fault segments and likely triggered the earthquake. Eyre *et al.* (*21*) estimated via numerical modeling an aseismic moment release for this event of about 0.9×10^{14} Nm (see figure 5d in (*21*)). The total injected volume concerning all the hydraulic fracturing stages before the earthquake happened, that is, until stage 23, is 28408 m^3 (table S1 in (*21*)). It is important to note that, unlike all other previous cases in our dataset, here fault slip has not been measured nor observationally inferred. Although the numerical model from which we extract the moment release assimilates a substantial body of geological, geophysical, and experimental data, besides reproducing the timing and magnitude of the mainshock, we believe the degree of uncertainty for the moment release in this case is higher than in other cases were observational constraints of fault slip are available. For this reason, the data point corresponding to this event is differentiated in Figs. 5 and 6 with respect to other data points

by using a symbol with a dashed perimeter instead of a solid one. For the rupture run-out distance, we consider an uncertainty range between 200 m and 400 m. The maximum value is taken as the distance between the mainshock hypocenter and the hydraulic fracturing depth where the fractures presumably intersected the fault (see figure 4 in (*21*)). The minimum value is taken as the distance between the hydraulic fracturing depth and the end of the frictionally stable fault segment (shales) upward from the hydraulically stimulated reservoir (see figure S3 in (*21*)).

The 2013 hydraulic stimulation at the Rittershoffen geothermal site, France

In 2013, a hydraulic stimulation for the development of a deep geothermal reservoir was performed in the Rittershoffen geothermal field, in Northeastern France (*52*). A total volume of approximately 2600 m^3 of water (see figure 10 in (*85*)) was injected into the crystalline basement through the so-called GRT-1 well with a 640 m open-hole section located at depths between 1920 m and 2560 m. The hydraulic stimulation lasted for approximately 18 hours. The seismicity taking place during the injection operation illuminated a fault whose location and orientation are consistent with a major structure that experienced a substantial permeability enhancement as a result of the stimulation (*52*). The fault plane that fits well with the micro-seismicity is a patch of about $W = 200$ m $\times$ $L = 300$ m. Upon stopping the injection, seismicity ceased immediately. However, after 4 seismically quiescent days, a second swarm episode occurred in a nearby fault. Lengliné *et al.* (*52*) found that an aseismic slip of $\delta = 1$ cm (estimated via empirical scaling relations) in the first reactivated fault patch of 200 m $\times$ 300 m could explain via quasi-static stress transfer the triggering of the second swarm. Following their analysis, the corresponding aseismic moment release could be estimated as $\sim \mu\delta LW$. Considering $\mu = 20$ GPa (*52*), we obtain 1.2×10^{13} Nm. In their study, Lengliné *et al.* (*52*) attributed the delay of the second swarm to the so-called clock-advance effect in earthquake nucleation produced in this case by the aseismic-slip stress jump. Recently, Sáez and Lecampion (*24*) discussed an alternative scenario in which this delay could be associated with the continuous propagation of aseismic slip during the shut-in stage, without the need for any clock-advance effect. To give an uncertainty range for this event, we consider the results from the simplifying modeling analysis in (*24*), which gives an estimate of slip at the injection point of about 0.28 cm. Assuming a triangular distribution of slip over a circular patch of radius $R \approx 250$ m (*24*), we estimate the moment release as $(\pi/3)\mu\delta R^2 \approx 3.67 \times 10^{12}$ Nm. Finally, the rupture run-out distance is simply considered as 300 m which is approximately the furthest

distance of the seismicity cloud from the injection point (*52*). This assumes that all the seismicity is induced by aseismic slip surrounding possibly unstable patches hosting the micro-seismic events. Therefore, this may be regarded as a maximum. It seems not possible to constrain a minimum value for the rupture run-out distance for this case. It is important to mention that similarly to the previous case in Canada (*21*), fault slip here has not been measured nor observationally inferred. Neither a compelling geomechanical model that assimilates the available data has been built. We should thus consider this poorly constrained case with caution. Future studies will ideally provide better constraints for this hydraulic stimulation. Alternatively, as more cases of injection-induced aseismic ruptures with either direct measurements or observational inferences of slip and rupture extent become available, one may consider discarding this point from future datasets. As for the previous case (*21*), we highlight the large uncertainty for this event in the main text by using a symbol with a dashed perimeter instead of a solid one.

A note on the injected fluid volumes for hydraulic-fracturing cases

For the two hydraulic-fracturing cases in our dataset (*10,21*), we have assumed that the total fluid volume injected during the hydraulic fracturing operations is the relevant quantity for comparison with our scaling relations. However, it is expected that a fraction of the injected fluid fulfilling the hydraulic fractures does not enter the fault zone. Estimating this fraction remains challenging in practice. Nevertheless, if we assume that a relatively small fraction of the total injected volume, say 10 percent, reaches the fault zone, Figs. 5 and 6 indicate that our conclusions would remain unchanged. In such a scenario, the hydraulic fracturing data points would shift slightly to the left, and the upper limits (N and M factors) bounding the field cases would be only marginally affected.

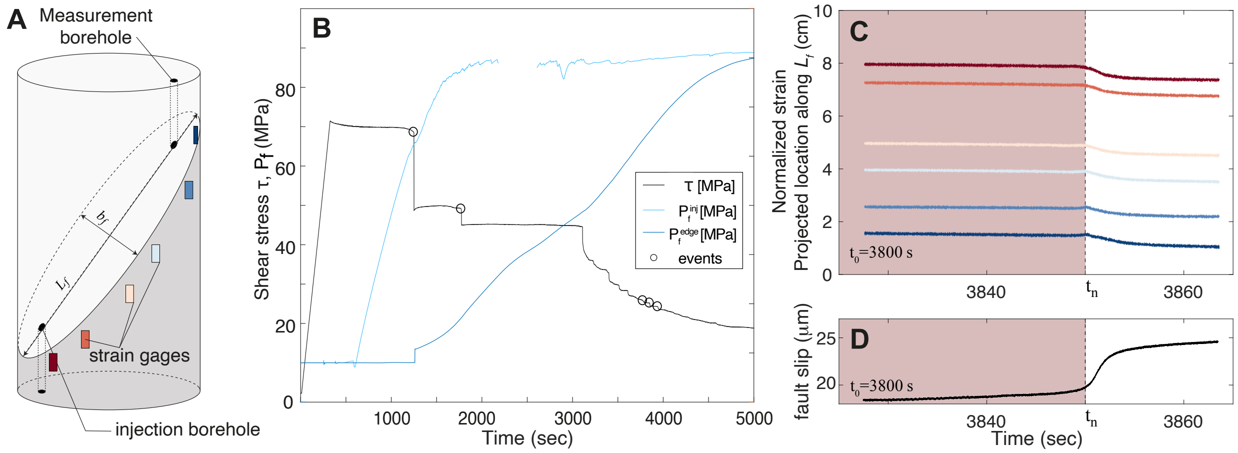

Figure S1: **Description of the centimeter-scale laboratory experiments.** (**A**) Schematic of the sample assembly. The length of the fault is $L_f = 8$ cm along strike. Injection is conducted in the bottom sample through a borehole reaching the fault interface. The evolution of the fluid pressure is measured both at the injection borehole and at the opposite edge of the fault through a measurement borehole. Strain gages are located along one side of the fault (see Passelègue *et al.* (*50*) for further details). (**B**) Evolution of the macroscopic shear stress (black solid line) and fluid pressure measured in the injection (cyan solid line) and measurement (blue solid line) boreholes during the experiments conducted at 95 MPa of confining pressure. Black circles correspond to aseismic slip events. (**C**) Evolution of the strain on each strain gage during the propagation of the slip front along the fault interface. The color code corresponds to the one used in panel (**A**) for the strain gages. t_n is the moment at which the slipping front reaches the edge of the sample and rigid block motion initiates, highlighted by the simultaneous release of stress on all strain gages. (**D**) Evolution of macroscopic fault slip during the propagation of an aseismic slip event.

Caption for Data S1. Dataset with estimates of moment release, rupture run-out distance, and injected fluid volumes. Excel file with the dataset used in Figs. 5 and 6. It is composed of two sheets named: "Fig. 5 - M0" and "Fig. 6 - R" containing the data for volume and moment release in Fig. 5, and volume and rupture run-out distance in Fig. 6, respectively.

REFERENCES AND NOTES

1. D. Hamilton, R. Meehan, Ground rupture in the baldwin hills. *Science* **172**, 333–344 (1971).

2. O. Scotti, F. Cornet, In situ evidence for fluid-induced aseismic slip events along fault zones. *Int. J. Rock. Mech. Min. Sci. Geomech. Abstr.* **31**, 347–358 (1994).

3. F. Cornet, J. Helm, H. Poitrenaud, A. Etchecopar, Seismic and aseismic slips induced by large-scale fluid injections. *Pure Appl. Geophys.* **150**, 563–583 (1997).

4. S. Bourouis, P. Bernard, Evidence for coupled seismic and aseismic fault slip during water injection in the geothermal site of Soultz (France), and implications for seismogenic transients. *Geophys. J. Int.* **169**, 723–732 (2007).

5. M. Calò, C. Dorbath, F. Cornet, N. Cuenot, Large-scale aseismic motion identified through 4-D P-wave tomography. *Geophys. J. Int.* **186**, 1295–1314 (2011).

6. Y. Guglielmi, F. Cappa, J. Avouac, P. Henry, D. Elsworth, Seismicity triggered by fluid injection-induced aseismic slip. *Science* **348**, 1224–1226 (2015).

7. S. Wei, J. P. Avouac, K. W. Hudnut, A. Donnellan, J. W. Parker, R. W. Graves, D. Helmberger, E. Fielding, Z. Liu, F. Cappa, M. Eneva, The 2012 Brawley swarm triggered by injection-induced aseismic slip. *Earth Planet. Sci. Lett.* **422**, 115–125 (2015).

8. F. Cappa, M. Scuderi, C. Collettini, Y. Guglielmi, J. Avouac, Stabilization of fault slip by fluid injection in the laboratory and in situ. *Sci. Adv.* **5**, eaau4065 (2019).

9. K. Materna, A. Barbour, J. Jiang, M. Eneva, Detection of aseismic slip and poroelastic reservoir deformation at the North Brawley Geothermal Field from 2009 to 2019. *J. Geophys. Res. Solid Earth* **127**, e2021JB023335 (2022).

10. T. Eyre, S. Samsonov, W. Feng, H. Kao, D. W. Eaton, InSAR data reveal that the largest hydraulic fracturing-induced earthquake in Canada, to date, is a slow-slip event. *Sci. Rep.* **12**, 2043 (2022).

11. K. S. Pepin, W. L. Ellsworth, Y. Sheng, H. A. Zebker, Shallow aseismic slip in the Delaware Basin determined by Sentinel-1 InSAR. *J. Geophys. Res. Solid Earth* **127**, e2021JB023157 (2022).

12. K. Im, J.-P. Avouac, On the role of thermal stress and fluid pressure in triggering seismic and aseismic faulting at the Brawley Geothermal Field, California. *Geothermics* **97**, 102238 (2021).

13. N. Z. Dvory, Y. Yang, E. M. Dunham, Models of injection-induced aseismic slip on height-bounded faults in the delaware basin constrain fault-zone pore pressure changes and permeability. *Geophys. Res. Lett.* **49**, e2021GL097330 (2022).

14. N. Deichmann, D. Giardini, Earthquakes induced by the stimulation of an enhanced geothermal system below Basel (Switzerland). *Seismol. Res. Lett.* **80**, 784–798 (2009).

15. W. Ellsworth, D. Giardini, J. Townend, S. Ge, T. Shimamoto, Triggering of the Pohang, Korea, earthquake (Mw 5.5) by enhanced geothermal system stimulation. *Seismol. Res. Lett.* **90**, 1844–1858 (2019).

16. M. B. Dusseault, M. S. Bruno, J. Barrera, Casing shear: Causes, cases, cures. *SPE Drill. Complet.* **16**, 98–107 (2001).

17. Y. Li, W. Liu, W. Yan, J. Deng, H. Li, Mechanism of casing failure during hydraulic fracturing: Lessons learned from a tight-oil reservoir in China. *Eng. Fail. Anal.* **98**, 58–71 (2019).

18. F. Cappa, J. Rutqvist, Modeling of coupled deformation and permeability evolution during fault reactivation induced by deep underground injection of CO_2. *Int. J. Greenhouse Gas Control* **5**, 336–346 (2011).

19. A. P. Rinaldi, V. Vilarrasa, J. Rutqvist, F. Cappa, Fault reactivation during CO_2 sequestration: Effects of well orientation on seismicity and leakage. *Greenh. Gases Sci. Technol.* **5**, 645–656 (2015).

20. P. Bhattacharya, R. Viesca, Fluid-induced aseismic fault slip outpaces pore-fluid migration. *Science* **364**, 464–468 (2019).

21. T. Eyre, D. W. Eaton, D. I. Garagash, M. Zecevic, M. Venieri, R. Weir, D. C. Lawton, The role of aseismic slip in hydraulic fracturing-induced seismicity. *Sci. Adv.* **5,** eaav7172 (2019).

22. V. Vilarrasa, S. De Simone, J. Carrera, A. Villaseñor, Unraveling the causes of the seismicity induced by underground gas storage at Castor, Spain. *Geophys. Res. Lett.* **48**, e2020GL092038 (2021).

23. A. Sáez, B. Lecampion, P. Bhattacharya, R. C. Viesca, Three-dimensional fluid-driven stable frictional ruptures. *J. Mech. Phys. Solids* **160**, 104754 (2022).

24. A. Sáez, B. Lecampion, Post-injection aseismic slip as a mechanism for the delayed triggering of seismicity. *Proc. R. Soc. A Math. Phys. Eng.* **479**, 20220810 (2023).

25. A. Sáez, B. Lecampion, Fluid-driven slow slip and earthquake nucleation on a slip-weakening circular fault. *J. Mech. Phys. Solids* **183**, 105506 (2024).

26. S. A. Shapiro, C. Dinske, J. Kummerow, Probability of a given-magnitude earthquake induced by a fluid injection. *Geophys. Res. Lett.* **34**, e2020GL092038 (2007).

27. S. A. Shapiro, O. S. Krüger, C. Dinske, C. Langenbruch, Magnitudes of induced earthquakes and geometric scales of fluid-stimulated rock volumes. *Geophysics* **76**, WC55–WC63 (2011).

28. A. McGarr, Maximum magnitude earthquakes induced by fluid injection. *J. Geophys. Res. Solid Earth* **119**, 1008–1019 (2014).

29. G. M. Atkinson, D. W. Eaton, H. Ghofrani, D. Walker, B. Cheadle, R. Schultz, R. Shcherbakov, K. Tiampo, J. Gu, R. M. Harrington, Y. Liu, M. van der Baan, H. Kao, Hydraulic Fracturing and Seismicity in the Western Canada Sedimentary Basin. *Seismol. Res. Lett.* **87**, 631–647 (2016).

30. N. van der Elst, M. Page, D. Weiser, T. Goebel, S. M. Hosseini, Induced earthquake magnitudes are as large as (statistically) expected. *J. Geophys. Res. Solid Earth* **121**, 4575–4590 (2016).

31. A. McGarr, A. J. Barbour, Wastewater disposal and the earthquake sequences during 2016 near Fairview, Pawnee, and Cushing, Oklohoma. *Geophys. Res. Lett.* **44**, 9330–9336 (2017).

32. M. Galis, J. Ampuero, P. Mai, F. Cappa, Induced seismicity provides insight into why earthquake ruptures stop. *Sci. Adv.* **3**, eaap7528 (2017).

33. L. De Barros, F. Cappa, Y. Guglielmi, L. Duboeuf, J.-R. Grasso, Energy of injection-induced seismicity predicted from in-situ experiments. *Sci. Rep.* **9**, 4999 (2019).

34. S. Bentz, G. Kwiatek, P. Martinez-Garzón, M. Bohnhoff, G. Dresen, Seismic moment evolution during hydraulic stimulations. *Geophys. Res. Lett.* **47**, e2019GL086185 (2020).

35. Z. Li, D. Elsworth, C. Wang, EGS-Collab, L. Boyd, Z. Frone, E. Metcalfe, A. Nieto, S. Porse, W. Vandermeer, R. Podgorney, H. Huang, T. McLing, G. Neupane, A. Chakravarty, P. J. Cook, P. F. Dobson, C. A. Doughty, Y. Guglielmi, C. Hopp, M. Hu, R. S. Jayne, S. E. Johnson, K. Kim, T. Kneafsey, S. Nakagawa, G. Newman, P. Petrov, J. C. Primo, M. Robertson, V. Rodriguez-Tribaldos, J. Rutqvist, M. Schoenball, E. L. Sonnenthal, F. A. Soom, S. Sprinkle, C. Ulrich, C. A. Valladao, T. Wood, Y. Q. Zhang, Q. Zhou, L. Huang, Y. Chen, T. Chen, B. Chi, Z. Feng, L. P. Frash, K. Gao, E. Jafarov, S. Karra, N. Makedonska, D. Li, J. Li, R. Pawar, N. Welch, P. Fu, R. J. Mellors, C. E. Morency, J. P. Morris, C. S. Sherman, M. M. Smith, D. Templeton, J. L. Wagoner, J. White, H. Wu, J. Moore, S. Brown, D. Crandall, P. Mackey, T. Paronish, S. Workman, B. Johnston, K. Beckers, J. Weers, Y. Polsky, M. Maceira, C. P. Chai, A. Bonneville, J. A. Burghardt, J. Horner, T. C. Johnson, H. Knox, J. Knox, B. Q. Roberts, P. Sprinkle, C. E. Strickland, J. N. Thomle, V. R. Vermeul, M. D. White, D. Blankenship, M. Ingraham, T. Myers, J. Pope, P. Schwering, A. Foris, D. K. King, J. Feldman, M. Lee, J. Su, T. Baumgartner, J. Heise, M. Horn, B. Pietzyk, D. Rynders, G. Vandine, D. Vardiman, T. Doe, J. McLennan, Y. S. Wu, J. Miskimins, P. Winterfeld, K. Kutun, M. D. Zoback, A. Singh, R. N. Horne, K. Li, A. Hawkins, Y. Zhang, E. Mattson, D. Elsworth, K. J. Im, Z. Li, C. J. Marone, E. C. Yildirim, J. Ajo-Franklin, A. Ghassemi, D. Kumar, V. Sesetty, A. Vachaparampil, H. F. Wang, H. Sone, K. Condon, B. Haimson, W. Roggenthen, C. Medler, N. Uzunlar, C. Reimers, M. W. McClure, Constraining maximum event magnitude during injection-triggered seismicity. *Nat. Commun.* **12**, 1528 (2021).

36. S. A. Shapiro, K.-H. Kim, J.-H. Ree, Magnitude and nucleation time of the 2017 Pohang Earthquake point to its predictable artificial triggering. *Nat. Commun.* **12**, 6397 (2021).

37. K. Im, J.-P. Avouac, E. R. Heimisson, D. Elsworth, Ridgecrest aftershocks at Coso suppressed by thermal destressing. *Nature* **595**, 70–74 (2021).

38. Y. Ida, Cohesive force across the tip of a longitudinal-shear crack and Griffith's specific surface energy. *J. Geophys. Res.* **77**, 3796–3805 (1972).

39. J. Caine, J. Evans, C. Forster, Fault zone architecture and permeability structure. *Geology* **24**, 1025–1028 (1996).

40. D. Faulkner, C. A. L. Jackson, R. J. Lunn, R. W. Schlische, Z. K. Shipton, C. A. J. Wibberley, M. O. Withjack, A review of recent developments concerning the structure, mechanics and fluid flow properties of fault zones. *J. Struct. Geol.* **32**, 1557–1575 (2010).

41. J. Marck, A. A. Savitski, E. Detournay, Line source in a poroelastic layer bounded by an elastic space. *Int. J. Numer. Anal. Methods Geomech.* **39**, 1484–1505 (2015).

42. E. Detournay, A. Cheng, "Fundamentals of poroelasticity" in *Analysis and Design Methods* (Elsevier, 1993), chap. 5, pp. 113–171.

43. D. H. Green, H. F. Wang, Specific storage as a poroelastic coefficient. *Water Resour. Res.* **26**, 1631–1637 (1990).

44. D. Garagash, L. Germanovich, Nucleation and arrest of dynamic slip on a pressurized fault. *J. Geophys. Res. Solid Earth* **117**, doi.org/10.1029/2012JB009209 (2012).

45. F. Ciardo, B. Lecampion. Injection-induced aseismic slip in tight fractured rocks. *Rock Mech. Rock Eng.* **56**, 7027–7048 (2023).

46. M. Parotidis, S. A. Shapiro, E. Rothert, Back front of seismicity induced after termination of borehole fluid injection. *Geophys. Res. Lett.* **31**, doi.org/10.1029/2003GL018987 (2004).

47. H. Kanamori, D. L. Anderson, Theoretical basis of some empirical relations in seismology. *Bull. Seismol. Soc. Am.* **65**, 1073–1095 (1975).

48. C. Segedin, Note on a penny-shaped crack under shear. *Math. Proc. Camb. Philos. Soc.* **47**, 396–400 (1951).

49. T. C. Hanks, H. Kanamori, A moment magnitude scale. *J. Geophys. Res. Solid Earth* **84**, 2348–2350 (1979).

50. F. X. Passelègue, M. Almakari, P. Dublanchet, F. Barras, J. Fortin, M. Violay, Initial effective stress controls the nature of earthquakes. *Nat. Commun.* **11**, 5132 (2020).

51. S. B. L. Cebry, C.-Y. Ke, G. C. McLaskey, The role of background stress state in fluid-induced aseismic slip and dynamic rupture on a 3-m laboratory fault. *J. Geophys. Res. Solid Earth* **127**, e2022JB024371 (2022).

52. O. Lengliné, M. Boubacar, J. Schmittbuhl, Seismicity related to the hydraulic stimulation of GRT1, Rittershoffen, France. *Geophys. J. Int.* **208**, ggw490–1715 (2017).

53. L. De Barros, G. Daniel, Y. Guglielmi, D. Rivet, H. Caron, X. Payre, G. Bergery, P. Henry, R. Castilla, P. Dick, E. Barbieri, M. Gourlay, Fault structure, stress, or pressure control of the seismicity in shale? Insights from a controlled experiment of fluid-induced fault reactivation. *J. Geophys. Res. Solid Earth* **121**, 4506–4522 (2016).

54. L. Duboeuf, L. de Barros, F. Cappa, Y. Guglielmi, A. Deschamps, S. Seguy, Aseismic motions drive a sparse seismicity during fluid injections into a fractured zone in a carbonate reservoir. *J. Geophys. Res. Solid Earth* **122**, 8285–8304 (2017).

55. D. R. Faulkner, T. M. Mitchell, E. Jensen, J. Cembrano, Scaling of fault damage zones with displacement and the implications for fault growth processes. *J. Geophys. Res. Solid Earth* **116**, doi.org/10.1029/2010JB007788 (2011).

56. H. M. Savage, E. E. Brodsky, Collateral damage: Evolution with displacement of fracture distribution and secondary fault strands in fault damage zones. *J. Geophys. Res. Solid Earth* **116**, doi.org/10.1029/2010JB007665 (2011).

57. X. Kuang, J. J. Jiao, C. Zheng, J. A. Cherry, H. Li, A review of specific storage in aquifers. *J. Hydrol.* **581**, 124383 (2020).

58. M. L. Doan, E. E. Brodsky, Y. Kano, K. F. Ma, In situ measurement of the hydraulic diffusivity of the active Chelungpu Fault, Taiwan *Geophys. Res. Lett.* **33,** doi.org/10.1029/2006GL026889 (2006).

59. L. Xue, H. B. Li, E. E. Brodsky, Z. Q. Xu, Y. Kano, H. Wang, J. J. Mori, J. L. Si, J. L. Pei, W. Zhang, G. Yang, Z. M. Sun, Y. Huang, Continuous permeability measurements record healing inside the wenchuan earthquake fault zone. *Science* **340**, 1555–1559 (2013).

60. J. Rutqvist, J. Noorishad, C.-F. Tsang, O. Stephansson, Determination of fracture storativity in hard rocks using high-pressure injection testing. *Water Resour. Res.* **34**, 2551–2560 (1998).

61. M. Almakari, H. Chauris, F. Passelègue, P. Dublanchet, A. Gesret, Fault's hydraulic diffusivity enhancement during injection induced fault reactivation: application of pore pressure diffusion inversions to laboratory injection experiments. *Geophys. J. Int.* **223**, 2117–2132 (2020).

62. K. F. Evans, A. Genter, J. Sausse, Permeability creation and damage due to massive fluid injections into granite at 3.5 km at Soultz: 1. Borehole observations. *J. Geophys. Res. Solid Earth* **110**, doi.org/10.1029/2004JB003168 (2005).

63. K. F. Evans, H. Moriya, H. Niitsuma, R. H. Jones, W. S. Phillips, A. Genter, J. Sausse, R. Jung, R. Baria, Microseismicity and permeability enhancement of hydrogeologic structures during massive fluid injections into granite at 3 km depth at the Soultz HDR site. *Geophys. J. Int.* **160**, 389–412 (2005).

64. A. McGarr, A. Barbour, Injection-induced moment release can also be aseismic. *Geophys. Res. Lett.* **45**, 5344–5351 (2018).

65. S. D. Goodfellow, M. H. B. Nasseri, S. C. Maxwell, R. P. Young, Hydraulic fracture energy budget: Insights from the laboratory. *Geophys. Res. Lett.* **42**, 3179–3187 (2015).

66. E. Detournay, Mechanics of hydraulic fractures. *Annu. Rev. Fluid Mech.* **48**, 311–339 (2016).

67. C. Marone, Laboratory-derived friction laws and their application to seismic faulting. *Annu. Rev. Earth Planet. Sci.* **26**, 643–696 (1998).

68. F. Ciardo, B. Lecampion, Effect of dilatancy on the transition from aseismic to seismic slip due to fluid injection in a fault. *J. Geophys. Res. Solid Earth* **124**, 3724–3743 (2019).

69. E. M. Dunham, Fluid-driven aseismic fault slip with permeability enhancement and dilatancy. *Philos. Transact. A Math. Phys. Eng. Sci.* **382**, 20230255 (2024).

70. S. A. Shapiro, *Fluid-Induced Seismicity* (Cambridge Univ. Press, 2015).

71. T. Goebel, E. Brodsky, The spatial footprint of injection wells in a global compilation of induced earthquake sequences. *Science* **361**, 899–904 (2018).

72. K. Sirorattanakul, Z. E. Ross, M. Khoshmanesh, E. S. Cochran, M. Acosta, J. P. Avouac, The 2020 Westmorland, California earthquake swarm as aftershocks of a slow slip event sustained by fluid flow. *J. Geophys. Res. Solid Earth* **127**, e2022JB024693 (2022).

73. Y. Yukutake, K. Yoshida, R. Honda, Interaction between aseismic slip and fluid invasion in earthquake swarms revealed by dense geodetic and seismic observations. *J. Geophys. Res. Solid Earth* **127**, e2021JB022933 (2022).

74. R. Bürgmann, The geophysics, geology and mechanics of slow fault slip. *Earth Planet. Sci. Lett.* **495**, 112–134 (2018).

75. Y. Tanaka, A. Kato, T. Sugano, G. Fu, X. Zhang, M. Furuya, W. Sun, S. Okubo, S. Matsumoto, M. Honda, Y. Sugawara, I. Ueda, M. Kusaka, M. Ishihara, Gravity changes observed between 2004 and 2009 near the Tokai slow-slip area and prospects for detecting fluid flow during future slow-slip events. *Earth Planets Space* **62**, 905–913 (2010).

76. J. Nakajima, N. Uchida, Repeated drainage from megathrusts during episodic slow slip. *Nat. Geosci.* **11**, 351–356 (2018).

77. E. Warren-Smith, B. Fry, L. Wallace, E. Chon, S. Henrys, A. Sheehan, K. Mochizuki, S. Schwartz, S. Webb, S. Lebedev, Episodic stress and fluid pressure cycling in subducting oceanic crust during slow slip. *Nat. Geosci.* **12**, 475–481 (2019).

78. H. Carslaw, J. Jaeger, *Conduction of Heat in Solids* (Clarendon Press, ed. 2, 1959).

79. K. Aki, P. Richards, *Quantitative Seismology*. (University Science Books, ed. 2, 2002).

80. I. Sneddon, *Fourier Transforms: International Series in Pure and Applied Mathematics* (McGraw-Hill, 1951).

81. S. A. Shapiro, C. Dinske, C. Langenbruch, F. Wenzel, Seismogenic index and magnitude probability of earthquakes induced during reservoir fluid stimulations. *Lead. Edge* **29**, 304–309 (2010).

82. D. Garagash, Fracture mechanics of rate-and-state faults and fluid injection induced slip. *Philos. Transact. A Math. Phys. Eng. Sci.* **379**, 20200129 (2021).

83. G. Barenblatt, The mathematical theory of equilibrium cracks in brittle fracture. *Adv. Appl. Mech.* **7**, 55–129 (1962).

84. M. Cocco, S. Aretusini, C. Cornelio, S. B. Nielsen, E. Spagnuolo, E. Tinti, G. D. Toro, Fracture energy and breakdown work during earthquakes. *Annu. Rev. Earth Planet. Sci.* **51**, 217–252 (2023).

85. C. Baujard, A. Genter, E. Dalmais, V. Maurer, R. Hehn, R. Rosillette, J. Vidal, J. Schmittbuhl, Hydrothermal characterization of wells GRT-1 and GRT-2 in Rittershoffen, France: Implications on the understanding of natural flow systems in the rhine graben. *Geothermics* **65**, 255–268 (2017).
